# Association of substance use and other psychiatric disorders with all-cause and external-cause mortality in individuals given community sentences in Sweden: a national cohort study

**DOI:** 10.1016/j.lanepe.2023.100703

**Published:** 2023-08-01

**Authors:** Denis Yukhnenko, Nigel Blackwood, Paul Lichtenstein, Seena Fazel

**Affiliations:** a Department of Psychiatry, University of Oxford, Oxford, UK; b Institute of Psychiatry, Psychology, and Neuroscience, King’s College London, London, UK; c Department of Medical Epidemiology and Biostatistics, Karolinska Institutet, Solna, Sweden

**Keywords:** Premature mortality, Mortality, Suicide, Mental health, Substance misuse, Criminal justice, Risk factors

## Abstract

**Background:**

Consistently high rates of premature mortality have been reported in individuals who receive community sentences. However, few studies have explored potential modifiable risk factors for these rates, particularly mental health. We examined the association of substance use and other psychiatric disorders with all-cause and external-cause mortality in individuals convicted of a criminal offence and given a community sentence.

**Methods:**

We did a longitudinal cohort study of 109,751 individuals given community sentences in Sweden using population-based registers. We calculated mortality rates for all-cause and external-cause mortality, hazard ratios for the association between psychiatric disorders and mortality, and population attributable fractions to quantify the contribution of psychiatric disorders to mortality risk.

**Findings:**

During the follow-up, 5749 (5.2%) individuals died, including 2709 (2.5%) from external causes. Individuals with pre-existing substance use and other psychiatric disorders had an increased mortality risk from any cause (aHR = 2.28 [95% CI 2.15–2.42]) and from external causes (3.11 [2.85–3.40]) compared to individuals without known psychiatric or substance use disorders. Suicide was the most common cause of death in younger persons.

**Interpretation:**

In individuals given community sentences, substance use and other psychiatric disorders were associated with an increased risk of premature death with suicide being the leading cause of death. Community supervision represents an opportunity to provide sentenced individuals with access to evidence-based treatment targeting substance misuse and psychiatric disorders to prevent potentially preventable deaths.

**Funding:**

10.13039/100010269Wellcome Trust.

**Research in context**

**Evidence before this study**

We conducted a comprehensive search on PubMed, without language restrictions, from 1 January 1966 to 1 February 2023, to identify articles on mental health risk factors for mortality in adults given community sentences. Search terms included “risk AND mental health AND (mortality OR death) AND (community sentence OR probation).” The identified publications included two cohort studies that investigated general and cause-specific mortality in adults individuals given community sentences. The first study reported outcomes for a cohort of first-time offenders in Australia, including released prisoners and probationers. Contact with mental health services in 5-year period before the sentence was associated with increased all cause-mortality (adjusted hazard ratio [aHR] = 1.9, 95% CI: 1.3–2.7) and for injury and poisoning deaths (aHR = 2.7, 95% CI: 1.7–4.1). The second examined opioid-related mortality in a cohort of US probationers. This found 15-fold increased risk (361 per 100,000 vs 23 per 100,000 in general population). We did not identify investigations that examined the association between psychiatric disorders and mortality in a representative cohort of community sentenced persons.

**Added value of this study**

We used high-quality linked population datasets to estimate cause-specific mortality rates and examine the association between psychiatric disorders and mortality in individuals given community sentences. We additionally examined the effects of specific diagnostic categories and substance use comorbidity. Our results showed that both all-cause and external cause mortality had consistently elevated associations with individual psychiatric disorders, including after accounting for familial confounding using sibling controls. Substance use disorders and other psychiatric disorders were more strongly associated with external-cause mortality than all-cause mortality. Compared with other diagnoses, drug use disorder had the strongest association with both all-cause and external cause mortality.

**Implications of all the available evidence**

Substance use and psychiatric disorders increase the risk of premature mortality in community-sentenced individuals, with high rates for external causes including suicide. Community supervision presents an opportunity to treat these disorders and prevent premature death.

## Introduction

Community sentences are a heterogenous group of sanctions widely used in the criminal justice system as an alternative to incarceration.^1^ These sentences involve mandatory activities carried out in the community and can include probation supervision, unpaid work, curfew, treatment for substance misuse and psychiatric disorders, and other sanctions. In many countries, community-sentenced individuals comprise the majority of the total criminal justice population. By the end of 2020, in the US, 60% of the criminal justice population (or 3 million individuals) were under probation supervision, with the remainder in custody or on parole.^2^^,^^3^ In 2021, in Sweden, around 11,109 individuals were given a community sentence, 9481 were sentenced to prison, and 4591 individuals completed parole.^4^

In Nordic countries, community sentences are widely implemented and serve as an intermediary sanction between fines and imprisonment.^5^ They consist of different forms of conditional sentences, probation supervision, and community service. Probation is the most common community sentence in Sweden,^6^ typically entailing a trial period of three years, including one year of supervision. Probation can also be coupled with conditions, such as treatment (including for substance misuse), vocational training, and community service.^7^ A conditional sentence allows a sentenced individual to avoid a custodial sentence on the condition that they live what is described as ‘an orderly life’ (e.g. stable accommodation, no new criminal charges), typically during a 2-year period.^7^ Conditional sentences are not supervised. A conditional sentence can be combined with fines and include community service. Committing a new crime during any community sentence can result in revocation and imposition of another sentence, such as imprisonment.

Community sentences generally aim to reduce recidivism while at the same time reducing the use of imprisonment. They can also provide sentenced individuals with better access to healthcare and welfare services, thus reducing risk of potential adverse health outcomes. Despite this, governmental agencies consistently reported elevated rates of premature mortality in community-sentenced individuals. In the US, the mortality rates of individuals on probation have been estimated at more than 2 times higher than the general population.^8^ In Europe, most countries reported individuals serving community sentences having substantially higher mortality rates than those in prison.^9^ Suicide contributes significantly to this increased mortality. In England and Wales, for example, 34% of the 2415 deaths of individuals under community supervision were self-inflicted.^10^ However, because of the lack of longitudinal studies, it is unclear whether high self-inflicted mortality rates continue beyond the period of community supervision. Addressing this premature mortality risk is part of a wider public health approach to meet the needs of vulnerable and neglected populations, who are overrepresented in criminal justice.^11^

Given these high mortality rates, the identification of risk factors associated with adverse health outcomes is a priority. UK-based research suggested that key modifiable risk factors were substance use and other psychiatric disorders, which are common in the community-sentenced population.^12^ Such disorders are associated with increased mortality in the general population.^13^ High rates of these disorders have also been shown in a population of released prisoners in the Netherlands.^14^ However, few studies have examined the association between psychiatric disorders and mortality in individuals given community sentences. A recent US study reported a 15-fold increase in opioid-related deaths among individuals given community sentences compared to general population.^15^ The reported increase was associated with identified drug use but did not examine other psychiatric disorders. A recent meta-analysis^16^ of premature mortality in offenders identified only one cohort study of community-sentenced individuals, which did not report data on psychiatric disorders.^17^ Other studies are limited by a lack of diagnostic specificity, sample selection, and short follow-up periods restricted to the post-supervision period.^18^ In addition, some have not fully adjusted for important confounders, thus likely overestimating the contribution of psychiatric disorders.

In the present study, we sought to address three research questions. First, whether psychiatric disorders were associated with all-cause and external-cause mortality in a community sentenced population. Second, the extent to which any observed associations were explained by comorbid substance use disorder. Third, to estimate the population impact of identified risk factors on all-cause and external-cause mortality. We additionally examined siblings given community sentences with and without psychiatric disorders to investigate whether such disorders were associated with mortality after accounting for familial confounding (early environmental and genetic factors shared by siblings).

## Methods

We followed STROBE guidelines^19^ for the reporting of observational studies (see Appendix 1 for the checklist).

### Study setting

We linked the data from several longitudinal, nationwide Swedish registers: National Crime Register that contains information about criminal offences and convictions since 1973; National Patient Register that provides information about psychiatric diagnoses for individuals admitted to inpatient hospitals (since 1973) and outpatient care (since 2001); Migration Register, containing dates of migration to and from Sweden; Cause of Death Register, which includes information on dates and causes of dates since 1958; Multi-Generational Register, containing information about biological relationships for individuals living in Sweden since 1933; Longitudinal Integration Database for Health Insurance and Labour Market studies that include yearly estimations of income benefit reception, marital and employment status, and education since 1990. The data linkage was done using a unique personal identifier for national registers, which every resident and immigrant in Sweden possesses.^20^

The Regional Ethics Committee at the Karolinska Institutet approved the current study (2013/5:8). Written consent from participants was not required as the study was conducted on anonymised routinely collected population register data and received ethics approval on this basis.

### Participants

We included Swedish residents aged 18 and older who received any community sentence at any point from November 1, 1991, to December 31, 2013. The community sentences imposed were probation with community service, probation with contracted treatment, and conditional sentences with a requirement for community service. These sentences cover all sanctions served in the community in Sweden (as described by Chapters 27 and 28 of the Swedish Penal Code), except for post-custodial supervision and probation combined with a prison sentence.^21^ At the start of the follow-up period for each individual, we used the date when a community sentence came into force. If a given individual received several community sentences over the study period, then the index sentence was selected at random. This allowed us to include not only individuals who served their first community sentence but also those with a prior criminal record and several such sentences, and was therefore more representative of the total community sentenced population.

The study cohort did not contain individuals whose cases were appealed or dismissed, as they did not appear in the sentencing register. We additionally identified full siblings within the cohort using the Multi-Generational Register.

### Measures

We extracted sociodemographic information, criminal and medical history available at the start of the community sentence. The socio-demographic information included biological sex at birth, age, marital status, years of education, employment information, and receipt of income support. Sociodemographic information is updated in Swedish registers once a year. For each individual, we extracted their most recent record within a year before the start of their sentence.

We also recorded if an individual had been sentenced before, for any offence or a violent offence, and whether their index offence was violent. A violent offence was defined as homicide, assault, robbery, arson, any sexual offence, illegal threats, or intimidation. To account for whether an individual was previously sentenced to prison or community sanctions, we recorded any prior imprisonment. Covariates were chosen based on prior studies of mortality in individuals released from prison.^22^^,^^23^ As available criminal records started from 1973, all individuals have their full criminal history available from age 15 (age of criminal responsibility), except for those born before 1958.

Medical history included any psychiatric diagnosis received before the index sentence and a history of self-harm. We used a hierarchical approach to classify the main diagnostic categories in line with previous research using Swedish national registers.^22^ The hierarchy was: schizophrenia spectrum disorders, bipolar disorder, depression, and any anxiety disorder. Thus, if an individual had a diagnosis of schizophrenia and any other diagnoses, we classified that individual as having schizophrenia. If an individual did not have schizophrenia but had bipolar disorder and depression, we classified that individual as having bipolar disorder, and so on.

To explore the effects of comorbidity between psychiatric disorders, we also investigated alcohol use disorder, drug use disorder, personality disorder, attention-deficit hyperactivity disorder, and other developmental or childhood disorders. We did not use a hierarchical approach for these comorbidities but examined whether they were present or not. ICD codes for the psychiatric diagnoses are listed in Appendix 2. We additionally coded the substance use disorder category as having either alcohol use disorder, drug use disorder, or both. We excluded nicotine use disorder.

To examine the potential effects of recent medical and criminal history, we conducted additional analysis by only including the last 5 years of medical and criminal history in the model. We also used 5-year all-cause and external-cause mortality as outcome for this analysis.

### Missing data

0.7% of individuals within the cohort did not have demographic information and 4.1% did not have education data at baseline. A sensitivity analysis demonstrated that the results did not differ significantly if the missing data were imputed (Appendix 3). Thus, in the primary analysis, we did not replace missing data by imputation or other methods.

### Outcomes and censoring

The outcome was death after receiving a community sentence. The underlying and contributing (secondary) causes of death are coded according to ICD-10 based on death certificates issued by physicians or forensic doctors. We extracted both all-cause mortality data and mortality information separated by the underlying cause of death using ICD chapters. Within external-cause mortality (ICD-10 Chapter XX), we further examined deaths by traffic and non-traffic accidents, suicide, and homicide. In keeping with previous work, we included undetermined deaths (ICD-10: Y10–Y34) as suicides, since their exclusion would underestimate the actual rates.^22^^,^^24^

All individuals were followed up until their death, permanent emigration from Sweden, or end of follow-up (December 31, 2013).

### Statistical analysis

We calculated mortality rates as the number of deaths for a given cause per person-years at risk. We used Kaplan–Meier survival curves to examine the timing of post-sentence mortality in individuals given community sentences with and without substance use disorder, and individuals with and without any other psychiatric disorders. We tested proportional hazards assumptions by visually examining the Kaplan–Meier curves and Schoenfeld residuals diagrams.

To explore the association between individual psychiatric disorders and mortality, we fitted Cox proportional hazard models for each diagnosis investigated. To estimate the total effect of individual psychiatric disorders on mortality, we fitted models adjusted for age and sex. We then adjusted for sociodemographic and criminological factors. To examine whether unmeasured familial factors partially explained the association between psychiatric disorders and death, we fitted a fixed-effect Cox regression model^25^ to a cohort of full siblings given community sentences. The model was stratified by family, so each sibling within one family had the same baseline hazard. In order to assess the discriminative accuracy of psychiatric diagnoses in predicting mortality, we employed the concordance index (c-index) as a metric, which is equivalent to the area under a ROC curve.^26^ This was done for the Cox regression models, both with and without variables representing previous psychiatric diagnoses.

To test whether psychiatric disorders were differently associated with mortality in men compared to women, independent of measured covariates, we also performed a stratified analysis for the model adjusted for sociodemographic and criminological factors.

To estimate the effect of comorbid substance use on mortality, we selected the individuals with a given psychiatric diagnosis and comorbid substance use and compared the risk of death to those without the diagnosis. Additionally, we selected individuals with a given diagnosis without comorbid substance use and compared the risk of death to those without a psychiatric diagnosis. The difference in risk estimates between individuals with and without comorbidity relative to individuals without any diagnoses corresponds to the observed effect of having comorbid substance use on mortality.

To estimate the population effect of substance use and other psychiatric disorders on mortality, we calculated population attributable fraction (PAF). The PAF estimates the proportion of deaths that can be attributed to a given risk factor, assuming a causal association exists between exposure and outcome. PAFs should interpreted with caution and are likely to provide the maximum possible estimate of the effect of removing a risk factor entirely, which is not possible in practice. To calculate PAF and corresponding CIs, we used the model-based adjusted attributable fraction function for Cox proportional hazard models in *AF* package for R.^27^

As our study was exploratory and the main models were pre-specified, no multiplicity correction methods were employed.^28^ The analyses were done in R using *survival* package.^29^

### Role of the funding source

The funders of the study had no role in the study design, data collection, data analysis, data interpretation, or writing of the report.

## Results

We identified 109,751 individuals (94,221 men and 15,530 women), who received at least one community sentence in Sweden during the study period (see Appendix 4 for the selection flowchart). These individuals were followed up for 685,453 person-years after their index sentence (see Appendix 5 for survival curves). We identified 9439 full siblings from 4479 families, who had been given a community sentence (see Appendix 6 for sibling estimation of individual diagnoses).

Baseline sociodemographic and criminological information, psychiatric diagnoses, and follow-up data are presented in Table 1. From the total cohort, 34,918 (31.8%) individuals had prior substance use disorder diagnoses, and 31,748 (28.9%) individuals had other psychiatric diagnoses. A higher proportion of women in the cohort (61.4%) had been diagnosed with a substance use disorder or other psychiatric disorder compared with men (41.2%). Univariate associations between baseline characteristics and death are presented in Appendix 7, and some collinearity was found, particularly among psychiatric diagnoses reflecting comorbidities (Appendix 8).

**Table 1:** Baseline characteristics and follow-up data of adult individuals receiving community sentences.

	Men	Women	Total
Number of individuals	94,221 (85.8%)	15,530 (14.2%)	109,751 (100.0%)
**Baseline characteristics**			
Any prior conviction	75,264 (79.9%)	10,735 (69.1%)	85,999 (78.4%)
Prior conviction for a violent crime	37,227 (39.5%)	3095 (19.9%)	40,322 (36.7%)
Prior prison sentence	25,937 (27.5%)	2186 (14.1%)	28,123 (25.6%)
Violent index sentence	40,292 (42.8%)	4652 (30.0%)	44,944 (41.0%)
Median age at sentence	30 (IQR: 22–43)	35 (IQR: 24–45)	31 (IQR: 22–44)
Age groups			
18–24 years	33,048 (35.1%)	4065 (26.2%)	37,113 (33.8%)
25–39 years	30,774 (32.7%)	5417 (34.9%)	36,191 (33.0%)
≥40 years	30,399 (32.3%)	6048 (38.9%)	36,447 (33.2%)
Married or in a registered partnership	12,249 (13.0%)	2424 (15.6%)	14,673 (13.4%)
Employed	39,142 (41.5%)	5042 (32.5%)	44,184 (40.3%)
Highest level of education			
<9 yr	4937 (5.2%)	844 (5.4%)	5781 (5.3%)
9–11 yr	78,289 (83.1%)	12,403 (79.9%)	90,692 (82.6%)
≥12 yr	7145 (7.6%)	1739 (11.2%)	8884 (8.1%)
Recipient of income support	32,583 (34.6%)	7172 (46.2%)	39,755 (36.2%)
Any psychiatric disorder	38,807 (41.2%)	9539 (61.4%)	48,346 (44.1%)
Any psychiatric disorder (other than substance use)	24,570 (26.1%)	7178 (46.2%)	31,748 (28.9%)
Schizophrenia spectrum disorder	2930 (3.1%)	739 (4.8%)	3669 (3.3%)
Bipolar disorder	1019 (1.1%)	452 (2.9%)	1471 (1.3%)
Depression	7603 (8.1%)	2745 (17.7%)	10,348 (9.4%)
Anxiety disorder	7462 (7.9%)	2421 (15.6%)	9883 (9.0%)
Alcohol use disorder	18,690 (19.8%)	4278 (27.5%)	22,968 (20.9%)
Drug use disorder	16,714 (17.7%)	4550 (29.3%)	21,264 (19.4%)
Substance (drug or alcohol) use disorder	28,154 (29.9%)	6764 (43.6%)	34,918 (31.8%)
Personality disorder	3885 (4.1%)	1667 (10.7%)	5552 (5.1%)
Attention-deficit hyperactivity disorder	4076 (4.3%)	702 (4.5%)	4778 (4.4%)
Other developmental or childhood disorder	3974 (4.2%)	906 (5.8%)	4880 (4.4%)
History of self-harm or prior suicide attempts	7901 (8.4%)	2975 (19.2%)	10,876 (9.9%)
**Follow-up data**			
Person-years at risk	593,088.1	92,365.1	685,453.2
Follow-up time	5.5 (IQR: 2.7–8.8)	5.2 (IQR: 2.6–8.6)	5.4 (IQR: 2.7–8.8)
Time until death	4.9 (IQR: 2.4–8.5)	4.5 (IQR: 2.3–7.3)	4.8 (IQR: 2.4–8.4)
Median age at death	49.4 (IQR: 35.0–59.8)	49.9 (IQR: 39.2–58.2)	49.5 (IQR: 35.7–59.7)
Deaths during follow-up	5096 (5.4%)	653 (4.2%)	5749 (5.2%)
within 1 year	539 (0.6%)	75 (0.5%)	614 (0.6%)
within 3 years	1585 (1.7%)	214 (1.4%)	1799 (1.6%)
within 5 years	2582 (2.7%)	365 (2.4%)	2947 (2.7%)
Deaths from external causes during follow-up	2396 (2.5%)	313 (2.0%)	2709 (2.5%)
within 1 year	341 (0.4%)	48 (0.3%)	389 (0.4%)
within 3 years	874 (0.9%)	120 (0.8%)	994 (0.9%)
within 5 years	1324 (1.4%)	194 (1.2%)	1518 (1.4%)
Emigrated during follow-up	1901 (2.0%)	232 (1.5%)	2133 (1.9%)

Note: All individuals received their sentences in the period from November 1, 1991, to December 31, 2013. 573 men and 69 women have missing values for marital status, employment, and income support. 3557 men and 497 women have missing values for education.

**Fig. 1:**
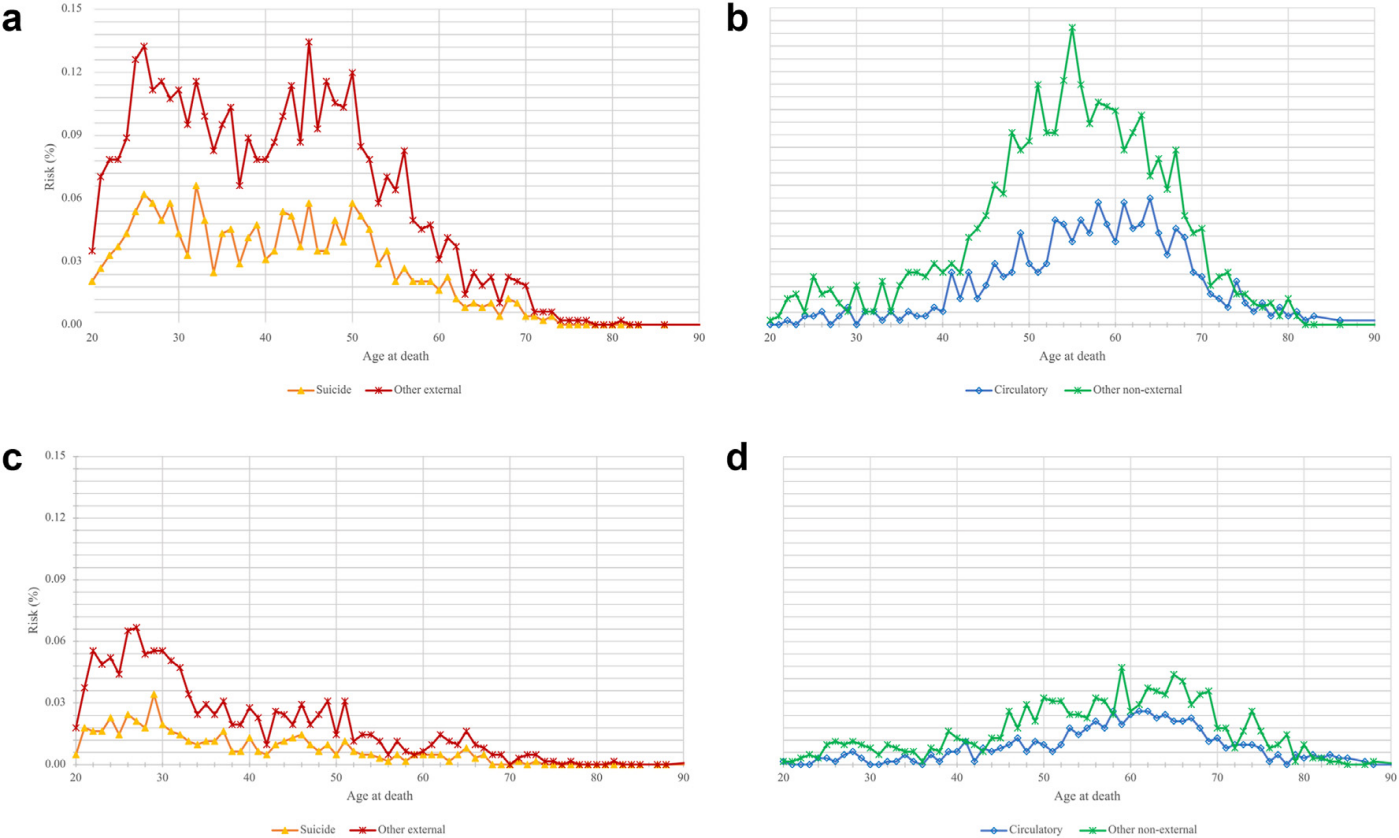
The absolute mortality risk in individuals given community sentences during the follow-up period by previous psychiatric disorder, cause of death, and age at death. a) risk of death in community sentenced individuals with psychiatric disorders (N = 48,436) from suicide or other external causes by age at death. b) risk of death in community sentenced individuals with psychiatric disorders (N = 48,436) from circulatory disorders or other non-external causes by age at death. a) risk of death in community sentenced individuals without psychiatric disorders (N = 61,405) from suicide or other external causes by age at death. b) risk of death in community sentenced individuals without psychiatric disorders (N = 61,405) from circulatory disorders or other non-external causes by age at death.

During follow-up, 5749 individuals died (Table 2) with 2709 deaths (47% of all deaths) having external causes. 1799 deaths (31%) occurred within 3 years after the sentence, which is the length of the probation supervision window. 614 (11%) deaths occurred within the first year and 2947 (51%) occurred within the first 5 years after the sentence. Overall, the all-cause mortality rate was 839 per 100,000 person-years and 395 per 100,000 person-years for external causes. The most common cause of death was suicide with 1170 (20%) deaths from the 5749 total. Cardiovascular disorders, traffic accidents, and cancer were other major causes of death. Most deaths from external causes occurred in younger persons, while deaths from other causes mostly occurred in people aged 50 and over, with diseases of the circulatory system being the leading cause in older adults (Fig. 1). In individuals aged 18–30, 961 deaths occurred during follow-up, 361 (38%) of which were suicides. Associations of psychiatric disorders with mortality from non-external causes are additionally reported in Appendix 9.

**Table 2:** Mortality rates in individuals given community sentences in Sweden.

Cause	Men		Women		Overall	
	No. deaths (%)	Mortality rate (95% CI)	No. deaths (%)	Mortality rate (95% CI)	No. deaths (%)	Mortality rate (95% CI)
All causes	5096 (100%)	859 (836–883)	653 (100%)	707 (653–761)	5749 (100%)	839 (817–860)
Certain infectious and parasitic diseases (chapter I)	129 (3%)	22 (18–26)	18 (3%)	19 (10–28)	147 (3%)	21 (18–25)
Neoplasms (chapter II)∗	575 (11%)	97 (89–105)	81 (12%)	88 (69–107)	656 (11%)	96 (88–103)
Endocrine, nutritional and metabolic diseases (chapter IV)	82 (2%)	14 (11–17)	14 (2%)	15 (7–23)	96 (2%)	14 (11–17)
Mental and behavioural disorders (chapter V)	221 (4%)	37 (32–42)	23 (4%)	25 (15–35)	244 (4%)	36 (31–40)
Diseases of the nervous system (chapter VI)	60 (1%)	10 (8–13)	3 (0%)	3 (0–7)	63 (1%)	9 (7–11)
Diseases of the circulatory system (chapter IX)	892 (18%)	150 (141–160)	103 (16%)	112 (90–133)	995 (17%)	145 (136–154)
Diseases of the respiratory system (chapter X)	163 (3%)	27 (23–32)	23 (4%)	25 (15–35)	186 (3%)	27 (23–31)
Diseases of the digestive system (chapter XI)†	354 (7%)	60 (53–66)	47 (7%)	51 (36–65)	401 (7%)	59 (53–64)
Symptoms, signs and abnormal clinical and laboratory findings, not elsewhere classified (chapter XVIII)	190 (4%)	32 (27–37)	21 (3%)	23 (13–32)	211 (4%)	31 (27–35)
Other non-external causes (chapters III, VII, VIII, XII-XVII)	34 (1%)	6 (4–8)	7 (1%)	8 (2–13)	41 (1%)	6 (4–8)
External causes of morbidity and mortality (chapter XX)	2396 (47%)	404 (388–420)	313 (48%)	339 (301–376)	2709 (47%)	395 (380–410)
Traffic accidents	210 (4%)	35 (31–40)	12 (2%)	13 (6–20)	222 (4%)	32 (28–37)
Non-traffic accidents	130 (3%)	22 (18–26)	11 (2%)	12 (5–19)	141 (2%)	21 (17–24)
Suicide	1004 (20%)	169 (159–180)	166 (25%)	180 (152–207)	1170 (20%)	171 (161–180)
Homicide	118 (2%)	20 (16–23)	9 (1%)	10 (3–16)	127 (2%)	19 (15–22)

Note: data are n (%) or mortality per 100,000 person-years (95% CI). Causes classified by ICD-10 chapters.

∗ Out of 1893 deaths caused by neoplasms, 556 (29%) were malignant neoplasms of digestive organs including 228 cases of malignant neoplasms of the liver.

† Out of 401 deaths caused by diseases of the digestive system, 356 (89%) were caused by alcoholic liver disease.

All-cause and external-cause mortality were differently associated with the type of community sentence. Compared to probation, a conditional sentence was associated with lower levels of all-cause mortality (HR = 0.38 [95% CI 0.35–0.41]) and external-cause mortality (HR = 0.29 [95% CI 0.25–0.33]) (Appendix 7).

For most individual psychiatric disorders, there were no significant differences in their association with all-cause or external-cause mortality between men and women (Appendix 10). The exception was alcohol use disorder, which had a stronger association with mortality in women than in men. Since the overall interaction effect between sex, psychiatric disorders, and mortality outcomes was negligible, further results are presented for the total cohort.

### All-cause mortality and psychiatric disorders

**Fig. 2:**
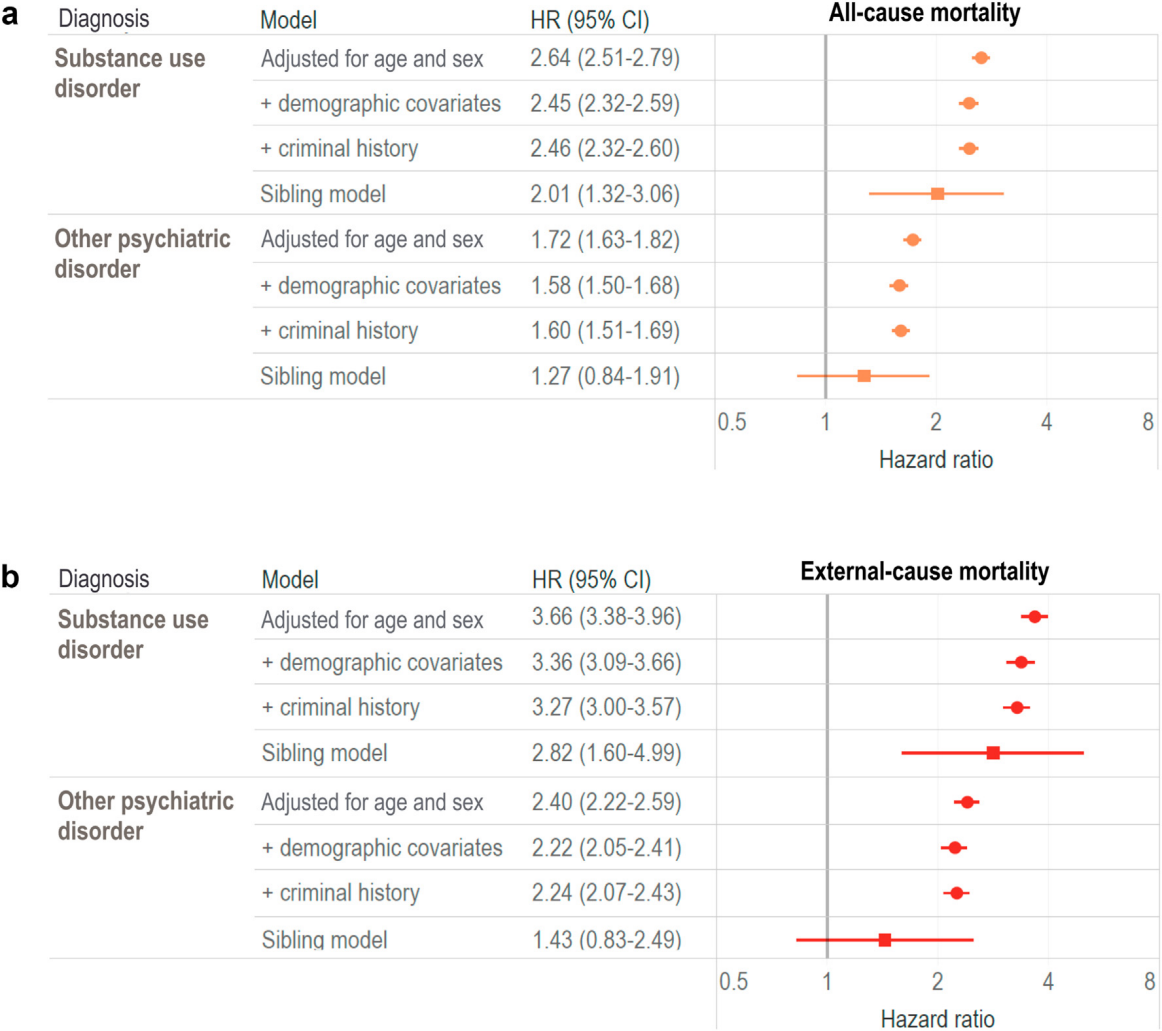
The association between all-cause mortality and external-cause mortality in individuals with substance use and other psychiatric disorders. a) the association between psychiatric disorders and all-cause mortality. b) the association between psychiatric disorders and external-cause mortality. Note: the initial model was adjusted for age and sex. Second model was additionally adjusted for sociodemographic covariates and third model was further adjusted for criminal history covariates. Sibling model adjusted for age and sex.

Individuals with prior diagnoses of substance use disorders were more likely to die during the follow-up period than individuals without substance use disorders (Fig. 2). The corresponding hazard ratio adjusted for age and sex was 2.64 (95% CI 2.51–2.79). This association remained significant after adjustment for other measured sociodemographic covariates and criminal history (Appendix 11 and Fig. 2A). In the sibling comparison model, adjusted for age and sex, the hazard ratio for the association between substance use and all-cause mortality was 2.01 (95% CI 1.31–3.06). Overall, assuming causality, 1531 of 5749 all deaths were potentially attributable to substance use, corresponding to a PAF of 26.6% (95% CI 24.5–28.8) (Appendix 11).

Having any psychiatric disorder other than substance use was also associated with an increased risk of death (Fig. 2A). The hazard ratio adjusted for age and sex was 1.72 (95% CI 1.63–1.82). Further adjustment for sociodemographic covariates and criminal history attenuated the association, but the estimates remained significant (Appendix 11 and Fig. 2A). The hazard ratio for the association between other psychiatric disorders and death estimated using the sibling model was 1.27 (95% CI 0.84–1.91). During the period of the study, assuming causality, 674 of all 3279 deaths were potentially attributable to psychiatric disorders other than substance use, which corresponds to a PAF of 12.4% (95% CI 11.0–13.8) (Appendix 12).

The associations with the all-cause mortality for individual psychiatric diagnoses other than substance use ranged from 1.47 (95% CI 1.36–1.60) for anxiety disorder to 2.38 (95% CI 2.02-2.80) for ADHD (Appendix 6). The associations between individual psychiatric diagnoses and all-cause mortality were attenuated after adjusting by comorbid substance use (Table 3).

**Table 3:** Association between mortality (all-cause and external cause) and substance use comorbidity in individuals given community sentences with prior psychiatric diagnosis.

	Incidence of death Died/No. individuals with disorder (%)		Hazard ratio (95% CI) Adjusted for age and sex	
	With substance use	Without substance use	With substance use	Without substance use
**Outcome: all-cause mortality**				
Any psychiatric (other than substance use)	3432/34,918 (10%)	449/13,428 (3%)	2.72 (2.57–2.88)	1.31 (1.18–1.45)
Schizophrenia spectrum	259/2528 (10%)	73/1141 (6%)	2.78 (2.44–3.17)	1.73 (1.37–2.18)
Bipolar	80/985 (8%)	26/486 (5%)	2.93 (2.34–3.67)	1.69 (1.15–2.49)
Depression	570/6495 (9%)	120/3853 (3%)	2.84 (2.58–3.13)	1.20 (0.99–1.44)
Anxiety	510/5419 (9%)	136/4464 (3%)	3.09 (2.81–3.41)	1.15 (0.97–1.37)
**Outcome: external-cause mortality**				
Any psychiatric (other than substance use)	1656/34,918 (5%)	239/13,428 (2%)	3.96 (3.63–4.32)	1.69 (1.46–1.96)
Schizophrenia spectrum	135/2528 (5%)	29/1141 (3%)	4.68 (3.88–5.64)	2.20 (1.52–3.19)
Bipolar	39/985 (4%)	18/486 (4%)	5.62 (4.04–7.80)	4.16 (2.60–6.65)
Depression	301/6495 (5%)	68/3853 (2%)	5.27 (4.58–6.06)	1.98 (1.54–2.54)
Anxiety	287/5419 (5%)	67/4464 (2%)	5.12 (4.46–5.88)	1.40 (1.09–1.80)

Note: hazard ratios were estimated by comparing individuals with psychiatric diagnoses to individuals without known psychiatric diagnoses.

The inclusion of substance use and other psychiatric disorders in the Cox regression model for all-cause mortality resulted in an improvement in discrimination. When adjusting for all measured covariates such as age, sex, sociodemographic variables, criminal history, and prior self-harm, the c-index improved by 6% (0.66–0.73) after also incorporating psychiatric disorder variables (Appendix 13).

### External cause mortality and psychiatric disorders

Individuals with previous diagnoses of substance use disorders were more likely to die from external causes during follow-up than individuals without substance use disorders (Fig. 2B). The corresponding hazard ratio adjusted for age and sex was 3.66 (95% CI 3.38–3.96). This association remained significant after adjustment for other measured sociodemographic covariates and criminal history (Appendix 11 and Fig. 2B). In the sibling model, adjusted for unmeasured familial confounding, the hazard ratio for the association between substance use and external-cause mortality was 2.82 (95% CI 1.60–4.99). Overall, assuming causality, 1136 of 2709 deaths from external causes were potentially attributable to substance use, which corresponds to a PAF of 42.0% (Appendix 12).

Having any psychiatric disorder other than substance use was also associated with an increased risk of death from an external cause (Fig. 2B). Hazard ratios remained significant after progressive adjustment for measured confounders (Appendix 11 and Fig. 2B), and for familial confounding using a sibling model (1.43 [95% CI 0.83–2.49]). During the study period, 704 of 2709 deaths were potentially attributable to psychiatric disorders other than substance use, which corresponds to the PAF of 26.0% (95% CI 23.5–28.5), assuming causality (Appendix 11).

Hazard ratios for individual diagnoses other than substance use ranged from 1.81 (95% CI 1.62–2.03) for anxiety disorder to 2.34 (95% CI 1.80–3.05) for bipolar disorder and 2.34 (95% CI 2.08–2.64) for personality disorder (Appendix 6). The associations between individual psychiatric diagnoses and external-cause mortality were attenuated after adjusting by comorbid substance use (Table 3).

The inclusion of substance use and other psychiatric disorders in the Cox regression model for external-cause mortality yielded improvements in discrimination. When adjusting for all measured covariates, such as age, sex, sociodemographic variables, criminal history, and prior self-harm, discriminative accuracy showed a 2% improvement (from c-index = 0.73 to c-index = 0.75) after incorporating psychiatric disorder variables (Appendix 13).

The results from imputed datasets did not significantly differ from the complete-case analysis. The analysis using the only the most recent 5-year criminal and medical history demonstrated higher association between psychiatric disorders and 5-year mortality outcomes than the primary analyses (Appendix 14).

## Discussion

We examined the association between psychiatric disorders and mortality in a Swedish nationwide population-based study of 109,751 individuals given community sentences over two decades. During follow-up, 5749 individuals died (all-cause mortality rate was 839 (95% CI 817–860) per 100,000 person-years). The leading cause of mortality was suicide (20%), with a rate of 171 (95% CI 161–180) per 100,000 person-years. Our study has three principal findings.

First, substance use and other psychiatric disorders were significantly associated with increased all-cause and external-cause mortality in individuals given community sentences. In non-sibling models, the association remained significant after adjustment for measured sociodemographic factors and prior criminal history. There was a slight attenuation in the sibling models, which could suggest either that substance misuse and other psychiatric disorders have common familial causes with mortality or that some effect of the psychiatric disorders is mediated through factors shared in the family.^30^ Certain genetic or familial risk factors for mortality have been shown to overlap with those for psychiatric disorders, such as those associated with increased risk of diabetes or cardiovascular diseases.^31^ Psychiatric disorders have also been associated with higher incidence of certain cancers,^32^ and this association could be influenced by risk factors such as smoking or drinking within a family. Furthermore, adjusting for comorbid substance misuse attenuated the association between other psychiatric disorders and mortality. This can be explained by substance use increasing the risk of fatal overdose by illicit and prescription drugs.^33^ Additionally, chronic substance use could lead to multi-organ damage thus increasing risk of non-external mortality, especially at older ages.^34^, ^35^, ^36^ Unmeasured or subthreshold substance misuse could also contribute to the association between identified psychiatric disorders and mortality. Several other pathways from psychiatric disorders to mortality, apart from those associated with substance use, are also plausible. Relevant psychosocial mechanisms could include engaging in antisocial lifestyle, developing antisocial attitudes and peer affiliations as well as having deficits in executive functions, including poor impulse control and emotional regulation.^37^^,^^38^ These increase the likelihood of engaging in risk-taking behaviours, including violent crime, that could lead to injury and death. Moreover, in our cohort of community-sentenced individuals, alcohol use disorder in women had a higher association with all-cause and external-cause mortality compared to men. This finding suggests potential sex-specific pathways in sentenced women with alcohol use, which warrant further investigation.

Second, substance use and other psychiatric disorders had stronger associations with external-cause mortality than with all-cause mortality. The number of potentially preventable deaths was particularly high in younger individuals (i.e., those under 35 years) with psychiatric disorders. This underscores the importance of psychiatric disorders as treatment targets in individuals given community sentences. Given the high prevalence and substantial health risks associated with substance use disorders, community-sentenced populations may benefit from interventions targeting substance misuse. Interventions include opiate substitution treatment, other anti-craving medications, and psychosocial interventions such as peer-support groups, contingency management, and specialised cognitive-behavioural therapy.^39^^,^^40^ In addition, there is evidence for the effectiveness of mental health interventions in people serving probation sententences.^41^ Identifying barriers that prevent sentenced individuals with psychiatric disorders from accessing available voluntary services should also be addressed. These can include mistrust of the healthcare system, low health literacy and help-seeking behaviour, fear of stigmatisation, and poor provision of services by healthcare providers.^42^ Another potentially modifiable factor for elevated mortality in individuals with psychiatric disorders is lower adherence to medication for physical health conditions.^43^

Third, suicide was the leading cause of death. Most suicides occurred in people in their late 20s. This 25-30 age group accounted for 238 (20%) out of the total of 1170 suicides during follow-up. In Sweden, in the general population, suicide rates ranged from 21 to 39 per 100,000 for men and from 8 to 17 per 100,000 for women aged 25–44 during the same period.^44^ Compared to these benchmark rates, suicide rates in community-sentenced individuals were at least 4 times higher for men and 11 times higher for women. Possible contributing factors for high suicide rates in community-sentenced individuals includes a higher prevalence of substance use and other psychiatric disorders, lower levels of social support, and adverse life events. Community supervision in itself can be a significant source of stress associated with a loss of control.^45^ Moreover, the deaths from external causes, including suicide, were not limited to the immediate post-sentence supervision period, which highlights the need for continuity of psychiatric care after sentencing. These findings suggest risk stratification could enhance decision-making as it is not feasible to offer gold standard assessments to all community-sentenced offenders. Such stratification needs to be linked to effective interventions, which will need to draw on interventions validated in the general population as there is no evidence base for suicide prevention interventions in those community-sentenced specifically.^46^ Such interventions can include early intervention for psychosis and other mental disorders, safety planning, and psychological therapies focusing on underlying mental health problems.^47^

The mortality rates obtained in our study were comparable to those estimated in released prisoners.^23^ The magnitude of the association between psychiatric disorders and mortality were commensurate with estimates from people leaving prison.^22^ This similarity in adverse health outcomes between individuals leaving prisons and those with community sentences suggests common risk markers and overlapping risk trajectories. Future research on trajectories could examine the effects of incident diagnoses, new sentences, and changes in socioeconomic variables in individuals with prior criminal histories using time-dependent methods. Another direction for future research is the examination of risk heterogeneity between subgroups of sentenced individuals using novel modelling approaches.^48^ Overall, in Nordic legal discussions, the emphasis has typically been on effective rehabilitation and prevention of reoffending, rather than the impact on health of community sentences. This study suggests that current practice should be reviewed in light of the negative health consequences of community sentences.

### Strength and limitations

We have examined risk factors using a large nationwide cohort with validated exposures and outcomes with sufficient power to examine individual diagnoses in the primary models and using sibling controls. Our estimates of the population effect of substance use and other psychiatric disorders on post-release mortality are also novel.

Our study has several limitations. We did not examine the effects of future sentences on healthcare trajectories. It is possible that individuals with substance use and other psychiatric diagnoses, having a higher risk of criminal recidivism, are more likely to go to prison at some point after being given community sentences. Controlling for potential future effects of imprisonment would allow for a stronger causal interpretation of the specific effects of community sentences on mortality risk. It has been demonstrated that multiple prison sentences are associated with higher mortality risk.^49^ In addition, a clear separation between the effects of custodial and non-custodial measures on mortality is another direction for research. Furthermore, as we did not formally account for multiple comparisons, caution is warranted for less consistent findings. Replication in new samples is an important next step.

Furthermore, we did not have information about the proportion of cases that were appealed or dismissed, and whether these persons were different in their prognosis. Based on data from 2014 to 2020, in Sweden, around 30% of community sentences were appealed.^50^ Therefore, generalisation to all recently sentenced individuals may be problematic, as it was unclear whether those who appealed successfully were different from those that served their sentence. To facilitate generalisability, we did not use variables that might be more predictive of the socioeconomic status in young people, such as parental income or education. Therefore, the measured sociodemographic covariates in younger people might play a lesser role in the primary analyses. However, sibling analyses adjusts for familial variables by design.

We used healthcare register data as a proxy for psychiatric disorders. The Swedish National Patient register does not contain any outpatient data recorded before 2001, which likely led to a conservative estimate of prevalence. This might potentially result in an overestimation of the association as severer cases were more likely to be identified in the patient register. Prior research demonstrated that individuals with schizophrenia or bipolar disorder are less prone to such bias.^51^ Moreover, our study was done in a single country that has a freely accessible public health system. Therefore, sentenced individuals may be more likely to access appropriate health interventions than in other countries, which might lead to conservative estimates of the effect of psychiatric disorders on mortality.

Another limitation is that sentencing practices and community sentencing definitions vary between countries.^52^ The variability may result in different legal selection criteria for individuals given community sentences, and, subsequently, different baseline risk levels. Some generalisability is suggested by the proportion of people sentenced to community probation with index violent offences, which is 22% in the US and 23% in Sweden.^53^^,^^54^ Another limitation is the lack of recent data and whether the use of such sentences has changed over time. However, official data does not suggest major changes in the last 30 years. The average annual number of community sentences has remained similar: 11,995 during 1993–2013, and 11,241 from 2014 to 2021. Furthermore, the proportion of all sentences that were community-based was 46% during the study period, which increased marginally to 50% during 2014–2021.^55^

### Conclusions

We have shown that substance use and other psychiatric disorders were associated with a higher risk of premature death in community-sentenced individuals. Most of these deaths were from external causes and potentially preventable. Suicide was the leading cause of death, disproportionally affecting younger individuals and individuals with psychiatric disorders. These findings underscore the importance of using community supervision as an opportunity for implementing evidence-based treatments targeting substance misuse and other psychiatric disorders in sentenced individuals.

## Contributors

SF was responsible for the conception of the study. Design and formal analysis were done by DY. DY, SF and PL had access to the data; DY accessed it and verified it with SF. DY drafted the paper, and SF, NB, PL reviewed, revised, and approved the final manuscript. SF provided overall supervision. All authors were responsible for the decision to submit for publication.

## Data sharing statement

The study was done using data from the Swedish population registers. The Public Access to Information and Secrecy Act in Sweden prohibits us from making individual-level data publicly available. Researchers interested in replicating our work can apply for individual-level data from Statistics Sweden (mikrodata@scb.se) for data from The Total Population Register (https://www.scb.se/vara-tjanster/bestallamikrodata/vilka-mikrodata-finns/individregister/registret-over-totalbefolkningen-rtb/), The Multi-Generation Register (https://www.scb.se/varatjanster/bestalla-mikrodata/vilka-mikrodata-finns/individregister/flergenerationsregistret/), and The Longitudinal Integrated Database for Health Insurance and Labour Market Studies (https://www.scb.se/en/services/guidance-for-researchersand-universities/vilka-mikrodata-finns/longitudinella-register/longitudinal-integrateddatabase-for-health-insurance-and-labour-marketstudies-lisa/); The National Board of Health and Welfare (registerservice@socialstyrelsen.se) for data from The National Patient Register (https://www.socialstyrelsen.se/patientregistret); and The Swedish National Council for Crime Prevention (statistik@bra.se) for data from The National Crime Register (https://www.bra.se/statistik/kriminalstatistik/specialbestallningar.html).

## Declaration of interests

We declare no competing interests.

## Appendix A Supplementary data

Appendix 1–14

Swedish Abstract
