# Supplementary material for: Association of substance use and other psychiatric disorders with all-cause and external-cause mortality in individuals given community sentences in Sweden: a national cohort study
Source: Lancet Reg Health Eur. 2023 Aug 1;33:100703. doi: 10.1016/j.lanepe.2023.100703 (PMC10636268; doi:10.1016/j.lanepe.2023.100703)
Supplement: Appendix 1–14 [file mmc1.docx]

**Appendix 1. STROBE reporting checklist (will be filled at the proof stage)**

**STROBE Statement—Checklist of items that should be included in reports of *cohort studies***

|  | Item No | Recommendation | Page  No. |
| --- | --- | --- | --- |
| **Title and abstract** | 1 | (*a*) Indicate the study’s design with a commonly used term in the title or the abstract | Title, Abstract |
|  |  | (*b*) Provide in the abstract an informative and balanced summary of what was done and what was found | Abstract |
| Introduction | | |  |
| Background/rationale | 2 | Explain the scientific background and rationale for the investigation being reported | 3-4 |
| Objectives | 3 | State specific objectives, including any prespecified hypotheses | 4 |
| Methods | | |  |
| Study design | 4 | Present key elements of study design early in the paper | 4-7 |
| Setting | 5 | Describe the setting, locations, and relevant dates, including periods of recruitment, exposure, follow-up, and data collection | 5 |
| Participants | 6 | (*a*) Give the eligibility criteria, and the sources and methods of selection of participants. Describe methods of follow-up | 5 |
|  |  | (*b*) For matched studies, give matching criteria and number of exposed and unexposed | NA |
| Variables | 7 | Clearly define all outcomes, exposures, predictors, potential confounders, and effect modifiers. Give diagnostic criteria, if applicable | 6-8 |
| Data sources/ measurement | 8* | For each variable of interest, give sources of data and details of methods of assessment (measurement). Describe comparability of assessment methods if there is more than one group | 5 |
| Bias | 9 | Describe any efforts to address potential sources of bias | 6-8 |
| Study size | 10 | Explain how the study size was arrived at | 5 |
| Quantitative variables | 11 | Explain how quantitative variables were handled in the analyses. If applicable, describe which groupings were chosen and why | 6, 8 |
| Statistical methods | 12 | (*a*) Describe all statistical methods, including those used to control for confounding | 7-8 |
|  |  | (*b*) Describe any methods used to examine subgroups and interactions | 7-8 |
|  |  | (*c*) Explain how missing data were addressed | 7 |
|  |  | (*d*) If applicable, explain how loss to follow-up was addressed | 7 |
|  |  | (*e*) Describe any sensitivity analyses | 6, 8 |
| Results | | |  |
| Participants | 13* | (a) Report numbers of individuals at each stage of study—eg numbers potentially eligible, examined for eligibility, confirmed eligible, included in the study, completing follow-up, and analysed | Appendix 4 |
|  |  | (b) Give reasons for non-participation at each stage | Appendix 4 |
|  |  | (c) Consider use of a flow diagram | Appendix 4 |
| Descriptive data | 14* | (a) Give characteristics of study participants (eg demographic, clinical, social) and information on exposures and potential confounders | Table 1 |
|  |  | (b) Indicate number of participants with missing data for each variable of interest | Appendix 7 |
|  |  | (c) Summarise follow-up time (eg, average and total amount) | Table 1, Appendix 5 |
| Outcome data | 15* | Report numbers of outcome events or summary measures over time | 9, Table 1, Appendix 5 |
| Main results | 16 | (*a*) Give unadjusted estimates and, if applicable, confounder-adjusted estimates and their precision (eg, 95% confidence interval). Make clear which confounders were adjusted for and why they were included | Appendix 7 |
|  |  | (*b*) Report category boundaries when continuous variables were categorized | Table 1, Appendix 7 |
|  |  | (*c*) If relevant, consider translating estimates of relative risk into absolute risk for a meaningful time period | Appendix 12 |
| Other analyses | 17 | Report other analyses done—eg analyses of subgroups and interactions, and sensitivity analyses | 12, Appendix 10 |
| Discussion | | |  |
| Key results | 18 | Summarise key results with reference to study objectives | 12-15 |
| Limitations | 19 | Discuss limitations of the study, taking into account sources of potential bias or imprecision. Discuss both direction and magnitude of any potential bias | 15-16 |
| Interpretation | 20 | Give a cautious overall interpretation of results considering objectives, limitations, multiplicity of analyses, results from similar studies, and other relevant evidence | 12-15 |
| Generalisability | 21 | Discuss the generalisability (external validity) of the study results | 16 |
| Other information | | |  |
| Funding | 22 | Give the source of funding and the role of the funders for the present study and, if applicable, for the original study on which the present article is based | 17 |

*Give information separately for exposed and unexposed groups.

Note: An Explanation and Elaboration article discusses each checklist item and gives methodological background and published examples of transparent reporting. The STROBE checklist is best used in conjunction with this article (freely available on the Web sites of PLoS Medicine at http://www.plosmedicine.org/, Annals of Internal Medicine at http://www.annals.org/, and Epidemiology at http://www.epidem.com/). Information on the STROBE Initiative is available at http://www.strobe-statement.org.

**Appendix 2. ICD codes for extracted variables.**

**Codes for prior psychiatric diagnoses and self-harm at baseline.**

| Diagnosis | Codes |
| --- | --- |
| Any psychiatric | ICD-8 codes: 290-315  ICD-9 codes: 290-319  ICD-10 codes: F00-F99 |
| Any psychiatric (excluding substance use diagnoses) | -\|\|- excluding codes for substance use |
| Schizophrenia spectrum disorder | ICD-8 codes: 295, 297, 298.1-9, 299  ICD-9 codes: 295, 297, 298 (ex. A), 299  ICD-10 codes: F20-F29 |
| Bipolar disorder | ICD-8 codes: 296.1, 296.3, 296.8  ICD-9 codes: 296A, 296C-E, 296W  ICD-10 codes: F30-F31 |
| Depressive disorder | ICD-8 codes: 296.2, 296.9, 298.0, 300.4  ICD-9 codes: 296B, 296X, 298A, 300E, 311  ICD-10 codes: F32-F39 |
| Anxiety disorder | ICD-8 codes: 300 (ex. .4), 305, 307  ICD-9 codes: 300 (ex. E), 306, 308, 309  ICD-10 codes: F40-F48 |
| Alcohol use disorder | ICD-8 codes: 291, 303  ICD-9 codes: 291, 303, 305A  ICD-10 codes: F10 |
| Drug use disorder | ICD-8 codes: 304  ICD-9 codes: 292, 304, 305 (ex. A)  ICD-10 codes: F11-F19 |
| Personality disorder | ICD-8 codes: 301  ICD-9 codes: 301 (ex. B)  ICD-10 codes: F60-F61 |
| Attention-deficit and hyperactivity disorder | ICD-9 codes: 314  ICD-10 codes: F90 |
| Other developmental disorder | ICD-8 codes: 308  ICD-9 codes: 299A, 312, 313, 315  ICD-10 codes: F80-F98 (ex. F90) |
| Self-harm | ICD-10 codes: X60-X69, X70-X79, X81-X84, Y10-Y19, Y20-Y29, Y30-Y34 |

**Appendix 3. Estimation of the association between substance use or other psychiatric disorders with all-cause and external-cause mortality in Cox regression models adjusted for sex, sociodemographic covariates, criminal history, and prior self-harm in imputed data.**

| Previous psychiatric disorder | Adjusted Cox regression models, HR (95% CI) | |
| --- | --- | --- |
| *Outcome* | ***All-cause mortality*** | ***External-cause mortality*** |
| Any psychiatric diagnosis | 2·10 (1·98-2·23) | 2·75 (2·52-3·01) |
| Any psychiatric diagnosis (other than substance use) | 1·44 (1·36-1·52) | 1·92 (1·77-2·09) |
| Schizophrenia spectrum | 1·24 (1·11-1·39) | 1·48 (1·25-1·74) |
| Bipolar | 1·30 (1·07-1·58) | 1·84 (1·41-2·41) |
| Depression | 1·22 (1·12-1·32) | 1·63 (1·45-1·83) |
| Anxiety | 1·31 (1·21-1·42) | 1·50 (1·34-1·68) |
| Personality disorder | 1·32 (1·21-1·44) | 1·61 (1·42-1·83) |
| Attention-deficit hyperactivity | 1·85 (1·57-2·19) | 1·74 (1·44-2·11) |
| Other developmental or childhood | 1·44 (1·26-1·64) | 1·41 (1·20-1·66) |
| Substance use | 2·28 (2·15-2·42) | 2·93 (2·69-3·20) |
| Alcohol use | 1·86 (1·76-1·97) | 1·97 (1·80-2·15) |
| Drug use | 2·02 (1·90-2·15) | 2·87 (2·63-3·12) |

Note: the data were imputed using Amelia package for R using all the covariates and the outcome as predictors. The 10 imputations were done. The resulting coefficients were then combined using Rubin’s rule (Rubin, D. Multiple imputation for nonresponse in surveys. 1987). sociodemographic factors included being employed, receiving income support, education level, marital status. Criminal history included any prior conviction, prior conviction for a violent crime, having a violent index offence. History of self-harm included hospitalisations and outpatient medical visits with ICD-10 codes X60-X84, Y10-Y34.

**Appendix 4. Selection process for the analysis cohort.**

**110,463** individuals given community sentences

during the period from November 1, 1991 to December 31, 2013

Excluded **712** individuals under 18 years old

Included **109,751** individuals

**105,357** individuals with full demographic information at sentencing

**9,439** full siblings (8,027 men; 1,412 women) from 4,479 families

Number of sentenced individuals under 18 years old excluded from the analysis in year each of the study:

| Year | No. | Year | No. |
| --- | --- | --- | --- |
| 1991 | 2 | 2003 | 70 |
| 1992 | 14 | 2004 | 71 |
| 1993 | 20 | 2005 | 59 |
| 1994 | 16 | 2006 | 50 |
| 1995 | 41 | 2007 | 27 |
| 1996 | 35 | 2008 | 19 |
| 1997 | 24 | 2009 | 36 |
| 1998 | 32 | 2010 | 30 |
| 1999 | 23 | 2011 | 13 |
| 2000 | 38 | 2012 | 17 |
| 2001 | 24 | 2013 | 11 |
| 2002 | 49 |  |  |

**Appendix 5. Kaplan-Meier curves for mortality in the cohort.**

**I. Total cohort survival. Outcome: all-cause mortality. Censoring: end of follow-up, emmigration.**


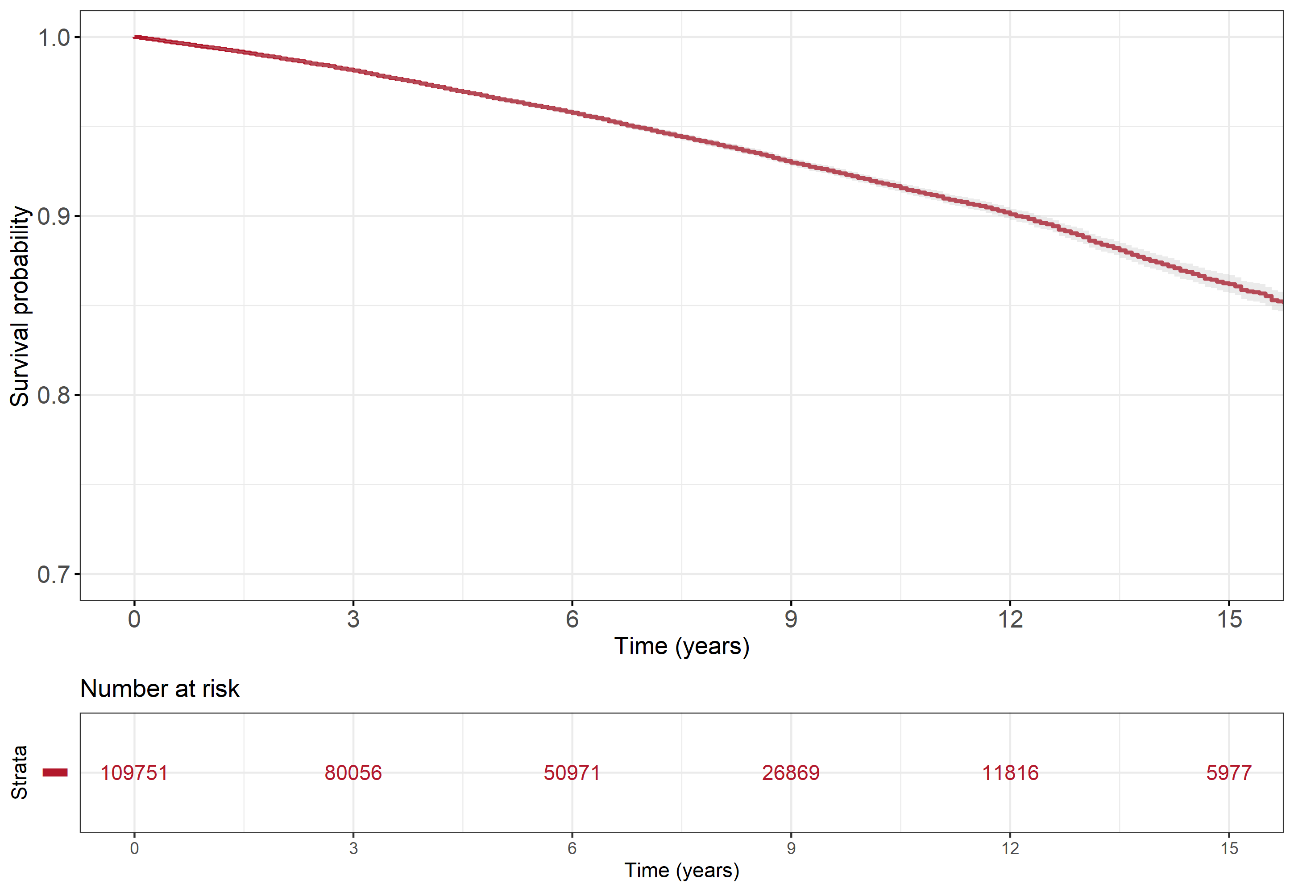


**II. Survival by age group. Outcome: all-cause mortality. Censoring: end of follow-up, emmigration.**


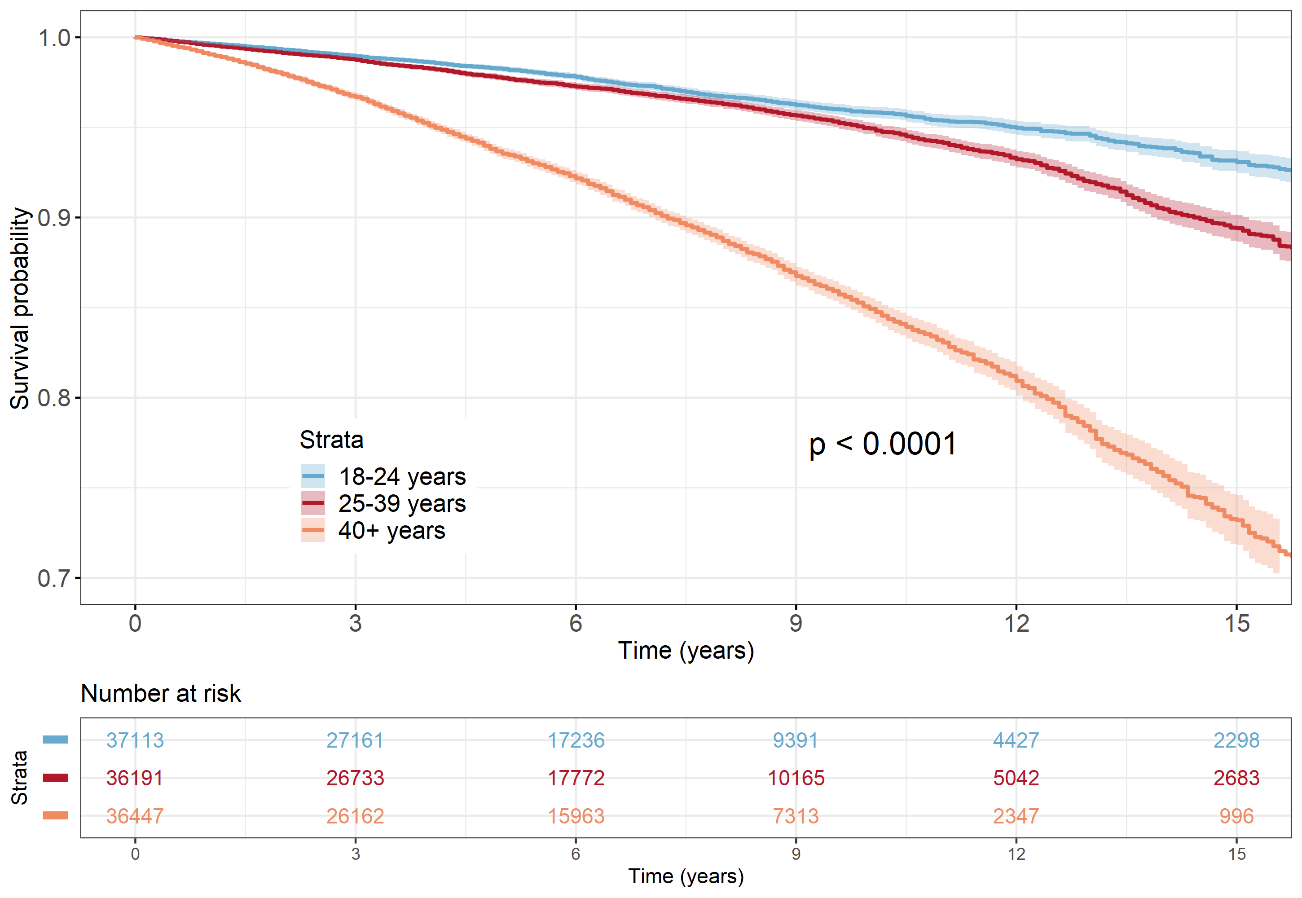


**III. Survival stratified by having substance use or other psychiatric diagnosis at sentence. Outcome: all-cause mortality. Censoring: end of follow-up, emmigration.**


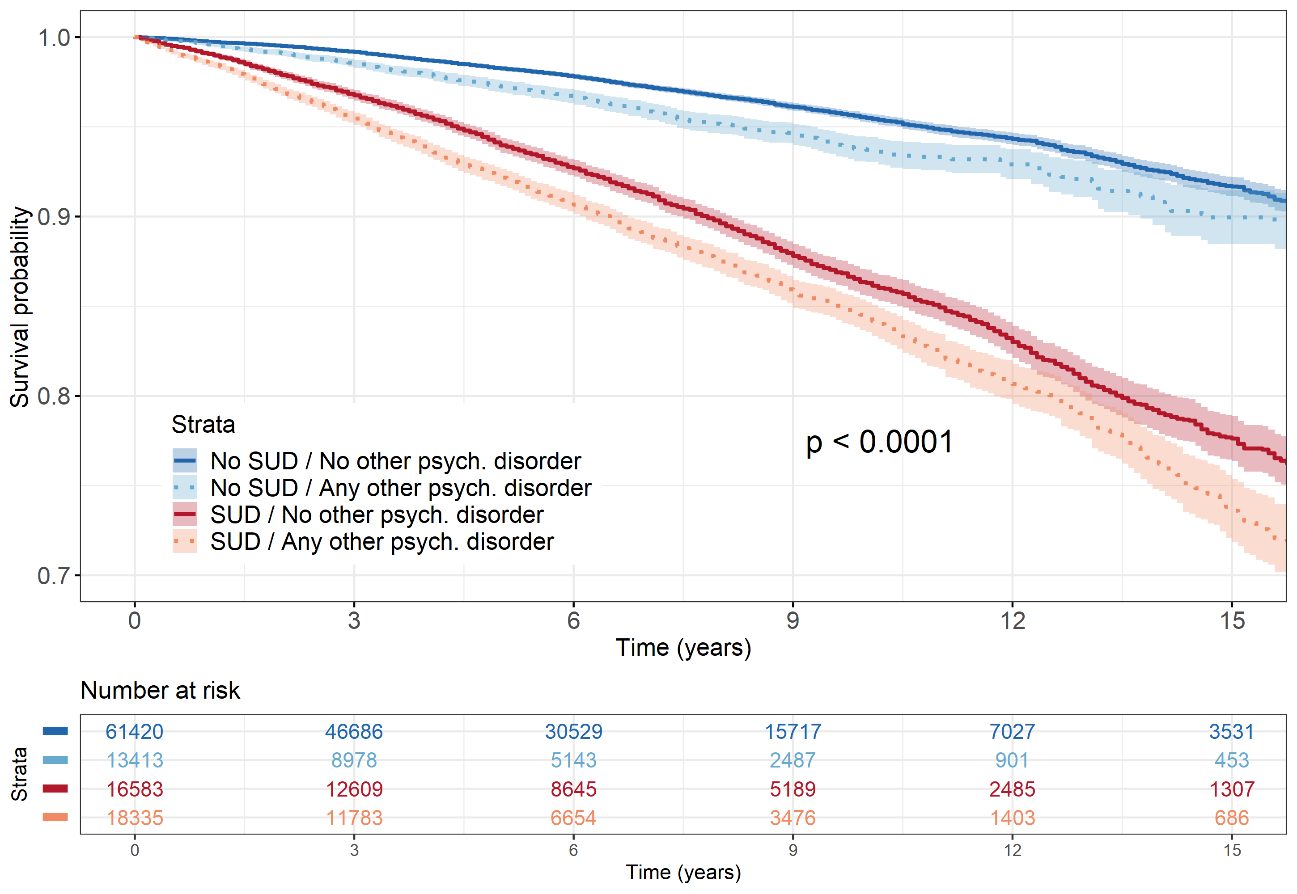


Note: SUD = substance use disorder (drug or alcohol use disorder).

**IV. Survival stratified by having substance use or other psychiatric diagnosis at sentence. Outcome: external-cause mortality. Censoring: end of follow-up, emmigration, death from non-external causes.**


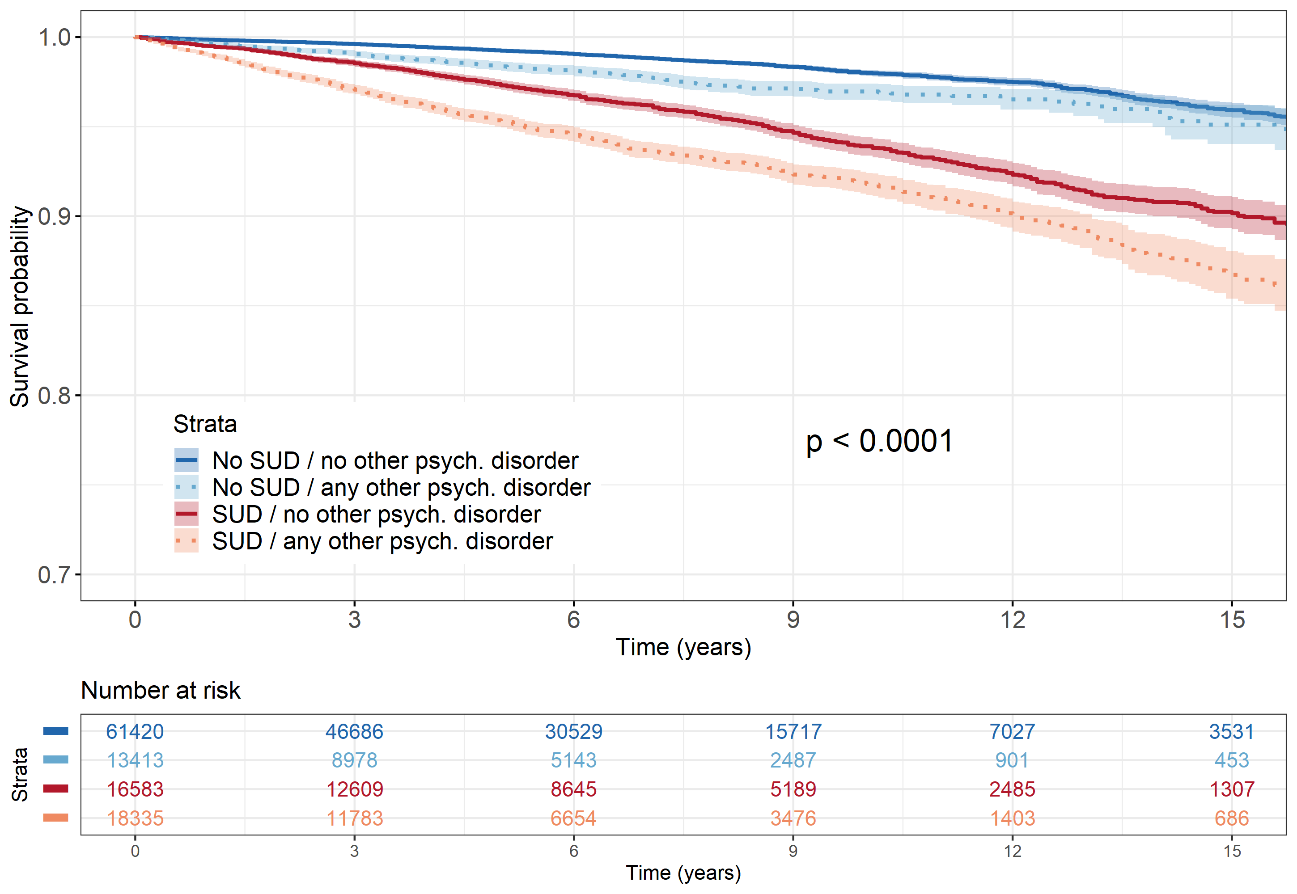


Note: SUD = substance use disorder (drug or alcohol use disorder).

**Appendix 6. Association between psychiatric disorders, prior self-harm and all-cause mortality in individuals given community sentences estimated by a fixed-effects sibling model. In these analyses, individuals with a given psychiatric disorder were compared to their siblings discordant by the diagnosis. The models were additionally adjusted for age and sex. We reported sibling models’ estimations only if there were at least 100 discordant siblings in the cohort.**

|  | Comparison between siblings | HR (95% CI) | |
| --- | --- | --- | --- |
| *Previous psychiatric disorder* | ***Cases / disc. siblings*** | ***All-cause*** | ***External cause*** |
| Any psychiatric diagnosis | 2,106 / 2,143 | 1·63 (1·08-2·46) | 2·19 (1·27-3·78) |
| Any psychiatric diagnosis (other than substance use) | 1,765 / 1,857 | 1·27 (0·84-1·91) | 1·43 (0·83-2·49) |
| Schizophrenia spectrum | 265 / 313 | 1·09 (0·45-2·67) | 0·98 (0·27-3·53) |
| Bipolar | 85 / 96 | - | - |
| Depression | 665 / 743 | 0·84 (0·44-1·60) | 1·18 (0·51-2·75) |
| Anxiety | 799 / 895 | 1·84 (1·04-3·28) | 1·99 (0·92-4·28) |
| Personality disorder | 448 / 509 | 0·89 (0·46-1·71) | 0·77 (0·32-1·85) |
| Attention-deficit hyperactivity | 401 / 438 | 0·86 (0·29-2·50) | 1·16 (0·36-3·75) |
| Other developmental or childhood | 419 / 471 | 0·98 (0·39-2·42) | 0·63 (0·12-3·17) |
| Substance use | 1,830 / 1,910 | 2·01 (1·32-3·06) | 2·82 (1·60-4·99) |
| Alcohol use | 1,291 / 1,382 | 1·61 (1·05-2·47) | 2·13 (1·22-3·71) |
| Drug use | 1,477 / 1,571 | 2·42 (1·59-3·69) | 3·43 (1·93-6·09) |
| Prior self-harm | 913 / 1,023 | 2·55 (1·52-4·29) | 3·46 (1·77-6·79) |

**Appendix 7. Association between baseline sociodemographic/clinical factors and mortality (all-cause and external cause) in individuals given community sentences. 739 individuals have missing values for marital status, employment, and income support. 4,394 individuals have missing values for education. Hazard ratios were not adjusted.**

| Total cohort N = 109,751 |  | All-cause mortality | |  | External cause mortality | |
| --- | --- | --- | --- | --- | --- | --- |
|  | N of individuals | N with outcome | Hazard ratio (95% CI) | N with outcome | | Hazard ratio (95% CI) |
| Age | |  |  |  | |  |
| 18-24 years | 37,113 | 1,013 (2·7%) | 1 | 861 (2·3%) | | 1 |
| 25-39 years | 36,191 | 1,443 (4·0%) | 1·36 (1·26-1·47) | 968 (2·7%) | | 1·09 (0·99-1·19) |
| ≥ 40 years | 36,447 | 3,293 (9·0%) | 3·93 (3·66-4·22) | 880 (2·4%) | | 1·19 (1·09-1·31) |
| Sex |  |  |  |  | |  |
| Male | 94,221 | 5,096 (5·4%) | 1·18 (1·09-1·29) | 2,396 (2·5%) | | 1·17 (1·04-1·32) |
| Female | 15,530 | 653 (4·2%) | 1 | 313 (2·0%) | | 1 |
| Civil status |  |  |  |  | |  |
| Single | 14,673 | 848 (5·8%) | 1·27 (1·18-1·37) | 230 (1·6%) | | 0·67 (0·59-0·77) |
| Married | 94,339 | 4,875 (5·2%) | 1 | 2,469 (2·6%) | | 1 |
| Highest education |  |  |  |  | |  |
| < 9 yr | 5,781 | 552 (9·5%) | 1 | 142 (2·5%) | | 1 |
| 9-12 yr | 8,884 | 444 (5·0%) | 0·69 (0·61-0·78) | 141 (1·6%) | | 0·83 (0·65-1·04) |
| > 12 yr | 90,692 | 4,555 (5·0%) | 0·53 (0·49-0·58) | 2,307 (2·5%) | | 1·04 (0·88-1·23) |
| Employed |  |  |  |  | |  |
| Yes | 44,184 | 1,479 (3·3%) | 0·64 (0·61-0·68) | 655 (1·5%) | | 0·58 (0·53-0·63) |
| No | 64,828 | 4,244 (6·5%) | 1 | 2,044 (3·2%) | | 1 |
| Recipient of income support |  |  |  |  | |  |
| Yes | 39,755 | 2,567 (6·5%) | 0·99 (0·94-1·04) | 1,449 (3·6%) | | 1·48 (1·37-1·60) |
| No | 69,257 | 3,156 (4·6%) | 1 | 1,250 (1·8%) | | 1 |
| Prior criminal history |  |  |  |  | |  |
| Any prior convictions | 85,999 | 5,093 (5·9%) | 1·46 (1·34-1·58) | 2,487 (2·9%) | | 2·20 (1·92-2·53) |
| No prior convictions | 23,752 | 656 (2·8%) | 1 | 222 (0·9%) | | 1 |
| Prior violent crime |  |  |  |  | |  |
| Prior conviction for a violent offence | 40,322 | 2,683 (6·7%) | 1·19 (1·13-1·26) | 1,344 (3·3%) | | 1·37 (1·27-1·48) |
| No prior convictions for a violent offence | 69,429 | 3,066 (4·4%) | 1 | 1,365 (2·0%) | | 1 |
| Prior prison |  |  |  |  | |  |
| Prior imprisonment | 28,123 | 2,581 (9·2%) | 1·76 (1·67-1·86) | 1,118 (4·0%) | | 1·56 (1·44-1·68) |
| No prior imprisonment | 81,628 | 3,168 (3·9%) | 1 | 1,591 (1·9%) | | 1 |
| Index violent crime |  |  |  |  | |  |
| Yes | 44,944 | 1,748 (3·9%) | 0·79 (0·75-0·84) | 877 (2·0%) | | 0·85 (0·79-0·92) |
| No | 64,807 | 4,001 (6·2%) | 1 | 1,832 (2·8%) | | 1 |
| Has pseudoreconviction (an unprosecuted offence) | | |  |  | |  |
| Yes | 13,942 | 955 (6·8%) | 0·98 (0·92-1·05) | 585 (4·2%) | | 1·40 (1·28-1·54) |
| No | 95,809 | 4,794 (5·0%) | 1 | 2,124 (2·2%) | | 1 |
| Any psychiatric disorder | |  |  |  | |  |
| Yes | 48,346 | 3,881 (8·0%) | 2·95 (2·79-3·12) | 1,895 (3·9%) | | 3·29 (3·03-3·57) |
| No | 61,405 | 1,868 (3·0%) | 1 | 814 (1·3%) | | 1 |
| Any psychiatric disorder (excl. substance use) | | |  |  | |  |
| Yes | 31,748 | 2,164 (6·8%) | 1·92 (1·82-2·02) | 1,161 (3·7%) | | 2·34 (2·17-2·52) |
| No | 78,003 | 3,585 (4·6%) | 1 | 1,548 (2·0%) | | 1 |
| Schizophrenia spectrum disorder |  |  |  |  | |  |
| Yes | 3,669 | 332 (9·0%) | 1·91 (1·71-2·13) | 164 (4·5%) | | 1·99 (1·70-2·34) |
| No | 106,082 | 5,417 (5·1%) | 1 | 2,545 (2·4%) | | 1 |
| Bipolar disorder |  |  |  |  | |  |
| Yes | 1,471 | 106 (7·2%) | 2·16 (1·78-2·62) | 57 (3·9%) | | 2·38 (1·83-3·09) |
| No | 108,280 | 5,643 (5·2%) | 1 | 2,652 (2·4%) | | 1 |
| Depressive disorder |  |  |  |  | |  |
| Yes | 10,348 | 690 (6·7%) | 1·85 (1·7-2) | 369 (3·6%) | | 2·07 (1·86-2·31) |
| No | 99,403 | 5,059 (5·1%) | 1 | 2,340 (2·4%) | | 1 |
| Anxiety disorder |  |  |  |  | |  |
| Yes | 9,883 | 646 (6·5%) | 1·53 (1·41-1·66) | 354 (3·6%) | | 1·79 (1·60-2·00) |
| No | 99,868 | 5,103 (5·1%) | 1 | 2,355 (2·4%) | | 1 |
| Alcohol use disorder |  |  |  |  | |  |
| Yes | 22,968 | 2,506 (10·9%) | 3·14 (2·98-3·31) | 1,017 (4·4%) | | 2·43 (2·25-2·63) |
| No | 86,783 | 3,243 (3·7%) | 1 | 1,692 (1·9%) | | 1 |
| Drug use disorder |  |  |  |  | |  |
| Yes | 21,264 | 2,128 (10·0%) | 2·41 (2·29-2·55) | 1,254 (5·9%) | | 3·56 (3·30-3·84) |
| No | 88,487 | 3,621 (4·1%) | 1 | 1,455 (1·6%) | | 1 |
| Substance use disorder (alcohol or drug use disorder) | | |  |  | |  |
| Yes | 34,918 | 3,432 (9·8%) | 3·29 (3·12-3·47) | 1,656 (4·7%) | | 3·49 (3·23-3·77) |
| No | 74,833 | 2,317 (3·1%) | 1 | 1,053 (1·4%) | | 1 |
| Personality disorder |  |  |  |  | |  |
| Yes | 5,552 | 594 (10·7%) | 2·07 (1·9-2·25) | 310 (5·6%) | | 2·33 (2·07-2·63) |
| No | 104,199 | 5,155 (4·9%) | 1 | 2,399 (2·3%) | | 1 |
| Attention deficit hyperactivity disorder | |  |  |  | |  |
| Yes | 4,778 | 152 (3·2%) | 1·42 (1·21-1·67) | 118 (2·5%) | | 2·20 (1·83-2·65) |
| No | 104,973 | 5,597 (5·3%) | 1 | 2,591 (2·5%) | | 1 |
| Other developmental or childhood disorder | |  |  |  | |  |
| Yes | 4,880 | 242 (5·0%) | 1·21 (1·06-1·38) | 118 (2·5%) | | 2·20 (1·83-2·65) |
| No | 104,871 | 5,507 (5·3%) | 1 | 2,591 (2·5%) | | 1 |
| Prior self-harm |  |  |  |  | |  |
| Yes | 10,876 | 880 (8·1%) | 2·32 (2·16-2·5) | 541 (5·0%) | | 3·12 (2·84-3·43) |
| No | 98,875 | 4,869 (4·9%) | 1 | 2,168 (2·2%) | | 1 |
| Immigrant | |  |  |  | |  |
| Yes | 23,334 | 828 (5·7%) | 0·64 (0·59-0·69) | 2,337 (2·7%) | | 0·6 (0·54-0·67) |
| No | 80,668 | 4,921 (3·4%) | 1 | 372 (1·5%) | | 1 |
| Type of community sentence | | | | | | |
| Conditional sentence | 39,811 | 774 (1·9%) | 0·38 (0·35-0·41) | 282 (0·7%) | | 0·29 (0·25-0·33) |
| Probation | 62,181 | 4941 (7·9%) | 1 | 2,410 (3·9%) | | 1 |

**Appendix 8. Pairwise collinearity between baseline covariates measured with Cramer’s V.**

Cramer’s V does not show the direction of associations, only the magnitude.

No association is 0, full collinearity is 1.


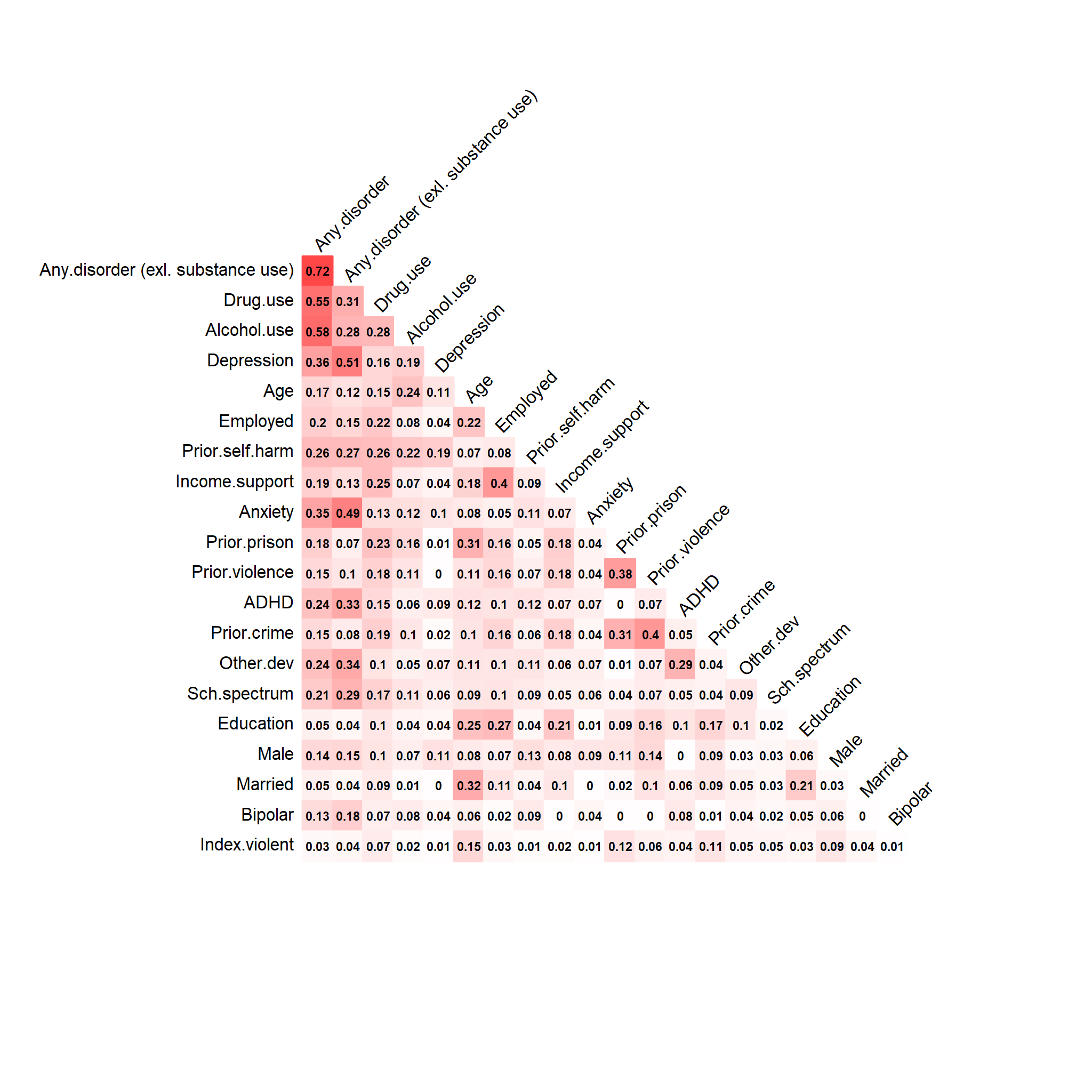


**Appendix 9. Estimation of the direct effect of individual psychiatric disorders on non-external cause mortality (ICD-10 chapters I-XVIII) relative to measured covariates.**

|  | Adjusted Cox regression models, HR (95% CI) | | | |
| --- | --- | --- | --- | --- |
| *Previous psychiatric disorder* | **Model 1**  Age, sex | **Model 2**  Age, sex, income support, employment, marital status, education | **Model 3**  Age, sex, income support, employment, marital status, education, prior crime, prior violent crime, index violent crime, prior imprisonment | **Model 4**  Age, sex, income support, employment, marital status, education, prior crime, prior violent crime, index violent crime, prior imprisonment, prior self-harm |
| Any psychiatric diagnosis | 2·03 (1·88-2·19) | 1·89 (1·75-2·05) | 1·86 (1·72-2·02) | 1·79 (1·65-1·94) |
| Any psychiatric diagnosis (other than substance use) | 1·37 (1·27-1·48) | 1·27 (1·17-1·37) | 1·26 (1·16-1·36) | 1·17 (1·08-1·27) |
| Schizophrenia spectrum | 1·43 (1·22-1·67) | 1·28 (1·09-1·50) | 1·28 (1·09-1·51) | 1·23 (1·05-1·44) |
| Bipolar | 1·18 (0·89-1·56) | 1·06 (0·80-1·42) | 1·07 (0·80-1·43) | 0·99 (0·74-1·32) |
| Depression | 1·13 (1·01-1·27) | 1·06 (0·94-1·19) | 1·06 (0·94-1·19) | 0·95 (0·85-1·08) |
| Anxiety | 1·30 (1·15-1·47) | 1·27 (1·12-1·43) | 1·25 (1·11-1·42) | 1·21 (1·07-1·37) |
| Personality disorder | 1·51 (1·33-1·70) | 1·34 (1·18-1·51) | 1·29 (1·14-1·47) | 1·20 (1·06-1·37) |
| Attention-deficit hyperactivity | 1·83 (1·30-2·58) | 1·56 (1·09-2·23) | 1·54 (1·07-2·20) | 1·41 (0·98-2·02) |
| Other developmental or childhood | 1·64 (1·31-2·06) | 1·53 (1·21-1·92) | 1·52 (1·21-1·91) | 1·38 (1·10-1·74) |
| Substance use | 2·20 (2·05-2·37) | 2·07 (1·92-2·23) | 2·03 (1·88-2·20) | 1·96 (1·81-2·12) |
| Alcohol use | 2·12 (1·97-2·27) | 2·01 (1·86-2·16) | 1·97 (1·82-2·12) | 1·90 (1·75-2·05) |
| Drug use | 1·93 (1·78-2·09) | 1·74 (1·60-1·90) | 1·70 (1·55-1·85) | 1·59 (1·45-1·74) |

**Appendix 10. Estimation of the direct effect of individual psychiatric disorders on all-cause and external-cause mortality relative to measured covariates stratified by sex.**

| *Previous psychiatric disorder* | *Men* | *Women* |
| --- | --- | --- |
| *Outcome: All-cause mortality* |  |  |
| Any psychiatric diagnosis | 2·08 (1·95-2·21) | 2·38 (1·93-2·93) |
| Any psychiatric diagnosis (other than substance use) | 1·42 (1·33-1·51) | 1·47 (1·24-1·75) |
| Schizophrenia spectrum | 1·23 (1·08-1·39) | 1·39 (1·04-1·87) |
| Bipolar | 1·37 (1·10-1·69) | 0·77 (0·45-1·32) |
| Depression | 1·21 (1·10-1·32) | 1·08 (0·88-1·33) |
| Anxiety | 1·35 (1·24-1·48) | 1·17 (0·95-1·44) |
| Personality disorder | 1·24 (1·12-1·37) | 1·71 (1·39-2·12) |
| Attention-deficit hyperactivity | 1·82 (1·52-2·18) | 1·67 (1·01-2·77) |
| Other developmental or childhood | 1·45 (1·25-1·68) | 1·36 (0·95-1·94) |
| Substance use | 2·24 (2·10-2·38) | 2·60 (2·17-3·11) |
| Alcohol use | 1·81 (1·70-1·92) | 2·37 (2·00-2·80) |
| Drug use | 2·00 (1·87-2·14) | 2·04 (1·71-2·44) |
|  |  |  |
| *Outcome: External-cause mortality* |  |  |
| Any psychiatric diagnosis | 2·68 (2·44-2·95) | 4·41 (3·01-6·48) |
| Any psychiatric diagnosis (other than substance use) | 1·87 (1·71-2·04) | 2·46 (1·89-3·20) |
| Schizophrenia spectrum | 1·42 (1·19-1·71) | 1·66 (1·12-2·47) |
| Bipolar | 1·99 (1·47-2·68) | 1·05 (0·54-2·05) |
| Depression | 1·62 (1·42-1·84) | 1·45 (1·10-1·91) |
| Anxiety | 1·57 (1·38-1·78) | 1·29 (0·97-1·72) |
| Personality disorder | 1·58 (1·37-1·82) | 1·70 (1·27-2·27) |
| Attention-deficit hyperactivity | 1·75 (1·42-2·15) | 1·37 (0·74-2·54) |
| Other developmental or childhood | 1·41 (1·17-1·69) | 1·32 (0·85-2·05) |
| Substance use | 2·86 (2·61-3·14) | 3·38 (2·54-4·50) |
| Alcohol use | 1·87 (1·70-2·05) | 2·63 (2·05-3·37) |
| Drug use | 2·84 (2·59-3·12) | 2·74 (2·12-3·54) |

Note: sociodemographic factors included being employed, receiving income support, education level, marital status. Criminal history included any prior conviction, prior conviction for a violent crime, having a violent index offence. History of self-harm included hospitalisations and outpatient medical visits with ICD-10 codes X60-X84, Y10-Y34.

**Appendix 11. Estimation of the direct effect of individual psychiatric disorders on all-cause and external-cause mortality relative to measured covariates.**

|  | Adjusted Cox regression models, HR (95% CI) | | |
| --- | --- | --- | --- |
| *Previous psychiatric disorder* | *Model 1.*  *Age + sex* | *Model 2.*  *Age + sex + sociodemographic factors* | *Model 3.*  *Age + sex + sociodemographic factors +*  *criminal history* |
| *Outcome: All-cause mortality* |  |  |  |
| Any psychiatric diagnosis | 2·47 (2·33-2·61) | 2·28 (2·15-2·41) | 2·28 (2·15-2·42) |
| Any psychiatric diagnosis  (other than substance use) | 1·72 (1·63-1·82) | 1·58 (1·50-1·68) | 1·60 (1·51-1·69) |
| Schizophrenia spectrum | 1·56 (1·40-1·74) | 1·35 (1·20-1·51) | 1·36 (1·21-1·53) |
| Bipolar | 1·56 (1·29-1·90) | 1·42 (1·17-1·74) | 1·44 (1·18-1·75) |
| Depression | 1·48 (1·36-1·60) | 1·39 (1·28-1·51) | 1·40 (1·29-1·52) |
| Anxiety | 1·47 (1·36-1·60) | 1·43 (1·31-1·55) | 1·42 (1·31-1·54) |
| Personality disorder | 1·75 (1·61-1·91) | 1·51 (1·39-1·65) | 1·50 (1·37-1·64) |
| Attention-deficit hyperactivity | 2·38 (2·02-2·80) | 2·11 (1·78-2·49) | 2·09 (1·77-2·48) |
| Other developmental or childhood | 1·82 (1·60-2·07) | 1·63 (1·42-1·86) | 1·63 (1·42-1·87) |
| Substance use | 2·64 (2·51-2·79) | 2·45 (2·32-2·59) | 2·46 (2·32-2·60) |
| Alcohol use | 2·20 (2·08-2·32) | 2·03 (1·92-2·15) | 2·03 (1·92-2·15) |
| Drug use | 2·46 (2·33-2·60) | 2·22 (2·09-2·35) | 2·23 (2·10-2·36) |
|  |  |  |  |
| *Outcome: External-cause mortality* |  |  |  |
| Any psychiatric diagnosis | 3·42 (3·15-3·72) | 3·17 (2·90-3·46) | 3·11 (2·85-3·40) |
| Any psychiatric diagnosis  (other than substance use) | 2·40 (2·22-2·59) | 2·22 (2·05-2·41) | 2·24 (2·07-2·43) |
| Schizophrenia spectrum | 1·96 (1·67-2·30) | 1·65 (1·40-1·95) | 1·68 (1·43-1·99) |
| Bipolar | 2·34 (1·80-3·05) | 2·15 (1·64-2·82) | 2·21 (1·68-2·89) |
| Depression | 2·09 (1·87-2·34) | 1·97 (1·76-2·21) | 2·01 (1·79-2·25) |
| Anxiety | 1·81 (1·62-2·03) | 1·73 (1·54-1·94) | 1·72 (1·53-1·93) |
| Personality disorder | 2·34 (2·08-2·64) | 2·00 (1·77-2·26) | 1·97 (1·74-2·24) |
| Attention-deficit hyperactivity | 2·32 (1·93-2·80) | 2·12 (1·75-2·57) | 2·10 (1·73-2·55) |
| Other developmental or childhood | 1·83 (1·56-2·14) | 1·64 (1·39-1·94) | 1·64 (1·38-1·94) |
| Substance use | 3·66 (3·38-3·96) | 3·36 (3·09-3·66) | 3·27 (3·00-3·57) |
| Alcohol use | 2·52 (2·32-2·73) | 2·28 (2·09-2·48) | 2·26 (2·08-2·47) |
| Drug use | 3·65 (3·38-3·93) | 3·33 (3·06-3·62) | 3·24 (2·97-3·52) |

Note: sociodemographic factors included being employed, receiving income support, education level, marital status. Criminal history included any prior conviction, prior conviction for a violent crime, having a violent index offence. History of self-harm included hospitalisations and outpatient medical visits with ICD-10 codes X60-X84, Y10-Y34.

**Appendix 12. Population attributable fraction of substance use and other psychiatric disorders for all-cause and external-cause mortality.**

|  | No. deaths | No. died with diagnosis | Adjusted hazard ratio (HR [95% CI]) | No. of deaths attributable to diagnosis | PAF  (% [95% CI]) |
| --- | --- | --- | --- | --- | --- |
| *Outcome: All-cause mortality* | | | | | |
| *Substance use disorder* | | | | | |
| Overall | 5,749 | 3,432 | 2·64 (2·51-2·79) | 1,531 | 26·6 (24·5-28·8) |
| Men | 5,096 | 2,960 | 2·62 (2·47-2·77) | 1,280 | 25·1 (22·9-27·3) |
| Women | 653 | 455 | 2·91 (2·46-3·44) | 257 | 39·4 (33·2-45·6) |
| *Other psychiatric disorder* |  |  |  |  |  |
| Overall | 5,749 | 2,164 | 1·72 (1·63-1·82) | 712 | 12·4 (11·0-13·8) |
| Men | 5,096 | 1,788 | 1·71 (1·62-1·81) | 566 | 11·1 ( 9·7-12·5) |
| Women | 653 | 364 | 1·80 (1·54-2·11) | 148 | 22·7 (16·7-28·7) |
|  |  |  |  |  |  |
| *Outcome: External-cause mortality* | | | | | |
| *Substance use disorder* | | |  |  |  |
| Overall | 2,709 | 1,656 | 3·66 (3·38-3·96) | 1,136 | 42·0 (39·2-44·7) |
| Men | 2,396 | 1,410 | 3·61 (3·32-3·93) | 961 | 40·1 (37·2-43·0) |
| Women | 313 | 239 | 4·33 (3·33-5·63) | 181 | 57·8 (49·6-66·1) |
| *Other psychiatric disorder* | | |  |  |  |
| Overall | 2,709 | 1,161 | 2·40 (2·22-2·59) | 704 | 26·0 (23·5-28·5) |
| Men | 2,396 | 943 | 2·32 (2·14-2·52) | 559 | 23·4 (20·8-25·9) |
| Women | 313 | 212 | 3·13 (2·46-3·98) | 151 | 48·1 (39·6-56·6) |

**Appendix 13. Predictive validity of Cox regression models for all-cause and external cause mortality with and without psychiatric disorders as covariates.**

**Concordance index (c-index) for multivariate Cox regression models adjusted for age, sex, income support, employment, marital status, education, prior crime, prior violent crime, index violent crime, prior imprisonment, prior self-harm and different combinations of psychiatric diagnosis cavariates.**

| Outcomes | Additional psychiatric diagnosis covariates | | | | |
| --- | --- | --- | --- | --- | --- |
|  | No mental health covariates | Any psychiatric disorder  (excl. drug or alcohol use) | Alcohol use disorder | Drug use disorder | Drug use disorder, alcohol use disorder, any psychiatric disorder |
| All-cause mortality | 0·728 | 0·742 | 0·740 | 0·752 | 0·762 |
| External-cause mortality | 0·668 | 0·702 | 0·683 | 0·715 | 0·735 |

**Сoncordance index of individual psychiatric disorders on all-cause and external-cause mortality in Cox regression models adjusted for age, sex, income support, employment, marital status, education, prior crime, prior violent crime, index violent crime, prior imprisonment, prior self-harm.**

| *Previous psychiatric disorder* | Adjusted Cox regression models, HR (95% CI) | |
| --- | --- | --- |
| *Outcome:* | *All-cause mortality* | *External-cause mortality* |
| Any psychiatric diagnosis | 0·749 | 0·713 |
| Any psychiatric diagnosis (other than substance use) | 0·733 | 0·692 |
| Schizophrenia spectrum | 0·726 | 0·666 |
| Bipolar | 0·726 | 0·665 |
| Depression | 0·727 | 0·672 |
| Anxiety | 0·728 | 0·668 |
| Personality disorder | 0·727 | 0·668 |
| Attention-deficit hyperactivity | 0·727 | 0·665 |
| Other developmental or childhood | 0·727 | 0·665 |
| Substance use | 0·752 | 0·711 |
| Alcohol use | 0·738 | 0·683 |
| Drug use | 0·744 | 0·704 |

Note: History of self-harm included hospitalisations and outpatient medical visits with ICD-10 codes X60-X84, Y10-Y34.

**Appendix 14. Estimation of the association between substance use or other psychiatric disorders and 5-year mortality in individuals given community sentences.**

|  | Adjusted Cox regression models, HR (95% CI) | | | | |
| --- | --- | --- | --- | --- | --- |
| *Previous psychiatric disorder* | **Model 1**  Age, sex | **Model 2**  Age, sex, income support, employment, marital status, education | **Model 3**  Age, sex, income support, employment, marital status, education, prior crime, prior violent crime, index violent crime, prior imprisonment | **Model 4**  Age, sex, income support, employment, marital status, education, prior crime, prior violent crime, index violent crime, prior imprisonment, prior self-harm | **Sibling model**  Age, sex |
| *All-cause mortality (5-year)* |  |  |  |  |  |
| Any psychiatric diagnosis | 3·10 (2·88-3·34) | 2·81 (2·59-3·04) | 2·82 (2·61-3·05) | 2·59 (2·38-2·81) | 2·05 (1·19-3·53) |
| Any psychiatric diagnosis  (other than substance use) | 2·25 (2·08-2·42) | 2·05 (1·89-2·21) | 2·10 (1·94-2·28) | 1·86 (1·71-2·02) | 1·39 (0·78-2·49) |
| Substance use | 3·17 (2·95-3·41) | 2·87 (2·66-3·10) | 2·86 (2·64-3·08) | 2·61 (2·41-2·83) | 2·63 (1·45-4·75) |
| Alcohol use | 2·46 (2·28-2·65) | 2·26 (2·09-2·45) | 2·27 (2·10-2·45) | 2·03 (1·87-2·20) | 1·84 (0·98-3·45) |
| Drug use | 3·17 (2·93-3·44) | 2·75 (2·53-3·00) | 2·74 (2·51-2·98) | 2·42 (2·22-2·65) | 2·96 (1·59-5·53) |
| *External-cause mortality (5-year)* |  |  |  |  |  |
| Any psychiatric diagnosis | 4·14 (3·71-4·62) | 3·72 (3·32-4·18) | 3·75 (3·35-4·21) | 3·31 (2·94-3·72) | 3·16 (1·55-6·44) |
| Any psychiatric diagnosis  (other than substance use) | 3·12 (2·82-3·46) | 2·85 (2·56-3·17) | 2·94 (2·64-3·27) | 2·51 (2·24-2·80) | 1·72 (0·84-3·55) |
| Substance use | 4·05 (3·65-4·49) | 3·60 (3·23-4·01) | 3·59 (3·22-4·01) | 3·13 (2·79-3·51) | 5·17 (2·16-12·36) |
| Alcohol use | 2·63 (2·35-2·94) | 2·35 (2·10-2·64) | 2·38 (2·12-2·67) | 1·99 (1·76-2·24) | 2·51 (1·12-5·64) |
| Drug use | 4·31 (3·89-4·78) | 3·81 (3·41-4·27) | 3·82 (3·41-4·28) | 3·27 (2·91-3·68) | 5·43 (2·23-13·2) |

Note: the medical and criminal history was extracted for 5 years prior to the index sentence.
