# Supplementary material for: Association of substance use and other psychiatric disorders with all-cause and external-cause mortality in individuals given community sentences in Sweden: a national cohort study
Source: Lancet Reg Health Eur. 2023 Aug 1;33:100703. doi: 10.1016/j.lanepe.2023.100703 (PMC10636268; doi:10.1016/j.lanepe.2023.100703)
Supplement: Swedish Abstract [file mmc2.docx]

**Abstract in Swedish**

This translation in Swedish was submitted by the authors and we reproduce it as supplied. It has not been peer reviewed. Our editorial processes have only been applied to the original abstract in English, which should serve as reference for this manuscript.

**Bakgrund**. Det har rapporterats om höga nivåer av förtida dödlighet hos personer som dömts till samhällstjänst. Få studier har dock undersökt potentiellt påverkbara riskfaktorer för förtida dödlighet, särskilt vad gäller faktorer relaterade till mental hälsa. Vi undersökte sambandet mellan psykiatriska eller beroendediagnoser och dödlighet av alla orsaker samt yttre orsaker hos personer som dömts till samhällstjänst.

**Metod.** Vi genomförde en longitudinell kohortstudie av 109 751 personer som dömts till samhällstjänst i Sverige med hjälp av populationsbaserade register. Vi beräknade dödlighetsfrekvenser för dödlighet av alla orsaker samt yttre orsaker, hazardkvoter för sambandet mellan psykiatriska och beroendediagnoser och dödlighet samt populationstillskrivna andelar för att kvantifiera bidraget från psykiatriska och beroendediagnoser på dödlighetsrisken.

**Resultat.** Under uppföljningsperioden avled 5 749 (5,2 %) personer, varav 2 709 (2,5 %) av yttre orsaker. Individer med befintliga psykiatriska och beroendediagnoser hade en ökad risk att dö av alla orsaker (aHR = 2,28 [95 % CI 2,15-2,42]) och yttre orsaker (3,11 [2,85-3,40]) jämfört med personer utan kända psykiatriska eller beroendediagnoser. Självmord var den vanligaste dödsorsaken bland yngre personer.

**Tolkning.** Hos personer som dömts till samhällstjänst var beroendediagnoser och andra psykiatriska diagnoser associerade med en ökad risk för förtida död, där självmord var den vanligaste dödsorsaken. Personer som döms till samhällstjänst bör få tillgång till evidensbaserad behandling riktad mot psykiatriska och beroendediagnoser för att minska risken för potentiellt förebyggbara dödsfall, särskilt självmord.

**Finansiering.**Wellcome Trust.
